# How ATP and dATP reposition class III ribonucleotide reductase cone domains to regulate enzyme activity

**DOI:** 10.1126/sciadv.ady9156

**Published:** 2025-11-28

**Authors:** Gisele A. Andree, Kelsey R. Miller-Brown, Zhuangyu Zhao, Ally K. Smith, Christopher D. Dawson, Daniel J. Deredge, Catherine L. Drennan

**Affiliations:** ^1^ Department of Chemistry, Massachusetts Institute of Technology, Cambridge, MA 02139, USA.; ^2^ Department of Biology, Massachusetts Institute of Technology, Cambridge, MA 02139, USA.; ^3^ Howard Hughes Medical Institute, Massachusetts Institute of Technology, Cambridge, MA 02139, USA.; ^4^ Department of Pharmaceutical Sciences, University of Maryland School of Pharmacy, Baltimore, MD 21201, USA.

## Abstract

Ribonucleotide reductases (RNRs) catalyze the conversion of ribonucleotides to deoxyribonucleotides. In the majority of cases, RNR activity is allosterically regulated by the cellular 2′-deoxyadenosine 5′-triphosphate (dATP)/adenosine 5′-triphosphate (ATP) ratio. To investigate allosteric activity regulation in anaerobic or class III (glycyl radical containing) RNRs, we determine cryo–electron microscopy structures of the class III RNR from *Streptococcus thermophilus* (StNrdD). We find that StNrdD’s regulatory “cone” domains adopt markedly different conformations depending on whether the activator ATP or the inhibitor dATP is bound and that these different conformations alternatively position an “active site flap” toward the active site (ATP-bound) or away (dATP-bound). In contrast, the position of the glycyl radical domain is unaffected by the cone domain conformations, suggesting that StNrdD activity is regulated through control of substrate binding rather than control of radical transfer. Hydrogen-deuterium exchange mass spectrometry and mutagenesis support the structural findings. In addition, our structural data provide insight into the molecular basis by which ATP and dATP binding lead to the observed differential cone domain conformations.

## Teaser

Structural snapshots reveal how ATP and dATP reposition class III RNR cone domains to control substrate binding and activity.

## INTRODUCTION

**Fig. 1.**
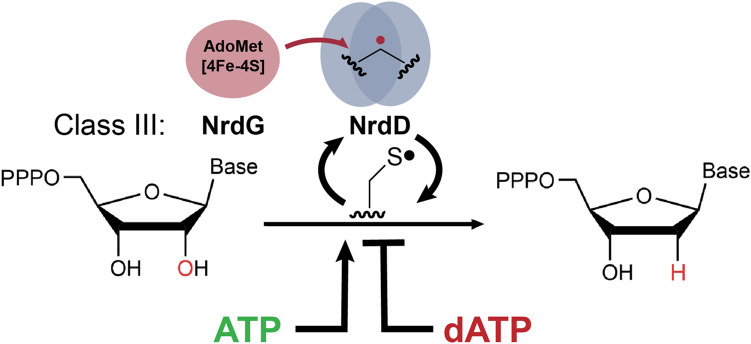
Reaction of an allosterically regulated class III RNR. Class III RNRs (NrdD) catalyze the reduction of ribonucleoside triphosphates to deoxyribonucleoside triphosphates using a catalytic thiyl radical generated via a glycyl radical species. This glycyl radical is installed on NrdD by a radical SAM activase (NrdG), which uses AdoMet and a [4Fe-4S] cluster. NrdD turnover also requires reducing equivalents, which are derived from formate in the case of the NrdD1 subclass. The activity of NrdD enzymes can be allosterically up-regulated by ATP and down-regulated by dATP.

Ribonucleotide reductases (RNRs) are essential enzymes that use radical-based chemistry to catalyze the reduction of ribonucleotides to deoxyribonucleotides (Fig. 1). RNRs have a central role in nucleotide metabolism, maintaining the balanced intracellular nucleotide pools that are needed for accurate DNA replication and repair (*1*–*5*). There are three main classes of RNRs (*2*, *6*, *7*). Each RNR class has a different tolerance or requirement for molecular oxygen and uses a different cofactor to generate a catalytically essential thiyl radical species in the active site (fig. S1) (*2*). Anaerobic class III RNRs (NrdDs), the subject of this study, use an oxygen-sensitive glycyl radical cofactor and are members of the glycyl radical enzyme (GRE) family (*8*, *9*). They are associated with both obligate and facultative anaerobes (*8*, *10*), including numerous microbial species that inhabit the human gut microbiome. They are found in pathogens, including *Staphylococcus aureus*, *Listeria monocytogenes*, *Shigella flexneri*, *Salmonella enterica*, and *Clostridioides difficile*. Differences between class III RNRs and the oxygen-dependent class Ia enzyme found in humans make class III RNRs attractive targets for the development of novel antibiotics. The development of new antibiotics is necessary, as antibiotic resistance is becoming a larger problem (*11*).

The glycyl radical cofactor is a simple but powerful catalyst. It is posttranslationally installed by a radical *S*-adenosylmethionine (SAM or AdoMet) activating enzyme, a NrdG in the case of class III RNRs (Fig. 1) (*9*, *12*, *13*). Installation involves a domain of the GRE, the glycyl radical domain (GRD) (fig. S2), flipping out of the enzyme core (*14*), and NrdG abstracting a hydrogen atom from a glycine residue to form the radical species (*12*, *15*). As a member of the radical SAM enzyme superfamily, NrdG requires a [4Fe-4S] cluster, AdoMet, and an electron for glycyl radical generation (*8*, *9*). After glycyl radical installation and the return of the GRD to the enzyme core, NrdD can catalyze numerous rounds of nucleoside triphosphate (NTP) reduction through the transient formation of the essential thiyl radical species (*8*). Although GREs are dimeric, glycyl radical content is typically less than two radicals per dimer, possibly due to half-of-sites reactivity (*8*).

To maintain the proper concentrations of deoxyribonucleotides in the cell and thereby DNA fidelity, RNRs are subject to allosteric regulation (*16*). Both the overall activity and the specificity toward a particular ribonucleotide substrate can be regulated (*2*). Specificity regulation involves the binding of a specificity effector to an allosteric site at the dimer interface (fig. S3A), which changes the preference of the enzyme for each of the four ribonucleotide substrates (*17*). Specificity effectors adenosine 5′-triphosphate (ATP) and dATP prompt cytidine 5′-triphosphate (CTP) and uridine 5′-triphosphate (UTP) reduction, thymidine 5′-triphosphate (TTP) prompts guanosine 5′-triphosphate (GTP) reduction, and 2′-deoxyguanosine 5′-triphosphate (dGTP) prompts ATP reduction (fig. S3B) (*17*–*19*). The mechanistic details of how specificity regulation happens in class III RNRs are not known. Activity regulation allows for the proper ratio of ribonucleotides to deoxyribonucleotides to be maintained in the cell with ATP up-regulating activity and dATP down-regulating enzyme activity (fig. S3B) (*2*). For the prototypical aerobic class Ia RNR from *Escherichia coli* (EcRNR), allosteric activity regulation involves dATP- and ATP-induced oligomeric state changes of its two subunits, the α_2_ catalytic subunit and the β_2_ radical-storage subunit (*20*, *21*). In particular, the binding of one dATP molecule to an N-terminal cone domain (site 1) of the α_2_ subunit results in formation of an inactive α_4_β_4_ state in which β_2_ is held away from α_2_, preventing radical transfer between the two subunits (fig. S4). Displacement of the dATP in site 1 by ATP, followed by the binding of a second ATP molecule in site 2 of the cone domain, destabilizes the α_4_β_4_ inactive state and shifts the equilibrium to the active α_2_β_2_ state that is capable of radical transfer (*21*). The molecular basis by which ATP binding destabilizes the α_4_β_4_ state of EcRNR has been recently established (*22*). Despite not requiring a β_2_ subunit, most class III RNRs are allosterically regulated by ATP and dATP, and about 76% of known class III RNRs contain one or multiple cone domains at their N termini (*23*). However, the molecular mechanism and conformational changes that accompany allosteric activity regulation in class III RNRs remain relatively unexplored.

On the basis of our knowledge of other RNR classes (*21*, *24*–*26*), two mechanisms for allosteric activity regulation in a class III RNR can be put forth. One involves GRD positioning, and the other involves the proper positioning of the substrate for radical-based catalysis. In analogy to EcRNR’s mechanism of holding the radical-storage β_2_ subunit at arm’s length when dATP is bound (fig. S4), dATP-bound cone domains could turn off the enzyme by holding the GRD too far from the thiyl radical–forming cysteine for short-range radical transfer (*21*). ATP binding could then reposition the GRD for radical transfer. Alternatively, dATP-bound cone domains could prevent substrate binding or hinder the proper positioning of substrate in a closed active site barrel for radical chemistry. In this latter case, ATP binding would allow the substrate to bind appropriately in a closed active site for catalysis. In EcRNR, the proper positioning of substrate for catalysis is regulated by the specificity effector exclusively, but the binding of the β_2_ subunit plays a role in sealing the active site (*21*, *24*, *27*). In general, radical chemistry requires a properly positioned substrate and a sealed active site to prevent uncoupling of radical transfer from catalysis. Thus, allosteric mechanisms that control radical transfer or control substrate binding/positioning in a closed active site can control RNR activity.

Insight into the mechanism of allosteric activity regulation in class III RNR was very recently provided by Logan and co-workers (*28*). These authors obtained cryo–electron microscopy (cryo-EM) structures of the cone domain–containing class III RNR from *Prevotella copri* (PcNrdD) (*28*). These structures are the first of a class III RNR that has cone domains. Authors solved structures in the presence of the allosteric activator ATP and also in the presence of allosteric inhibitor dATP (*28*). They observed an unexpected dimer-to-tetramer oligomeric state shift when dATP was present under the condition. In this dATP-inhibited tetrameric state, GRDs are disordered, no substrate is bound, and the active sites are open. In contrast, when ATP is bound to the cone domains, the enzyme is dimeric, the GRDs are ordered, substrate CTP is bound in one of the two active sites, and an “active site flap” covers the active site. This active site flap has an Asn-X-Asn sequence motif and is located C-terminal to the cone domain and N-terminal to the active site barrel. Briefly, differences were observed between ATP and dATP conditions with respect to both the GRD positioning and substrate binding, leaving both allosteric mechanisms described above on the table as potentially applicable.

To address the open questions about allosteric regulation of activity in class III RNRs, we present the first structures of the cone domain–containing class III RNRs from *Streptococcus thermophilus* (StNrdDs). *S. thermophilus* is a facultative anaerobe widely used in commercial dairy, in fermented products, and as a commercial probiotic supplement (*29*, *30*). It contains multiple RNR genes, including StNrdD, which is a member of the NrdD1 subclass of class III RNRs that uses formate as a source of reducing equivalents for ribonucleotide reduction (*8*). By using cryo-EM and x-ray crystallography, we obtain structures of StNrdD under allosterically activating (ATP) and inhibiting (dATP) conditions. In contrast to the PcNrdD structural data, we find that the GRD is ordered in the presence of both ATP and dATP and that the distance between the glycyl radical– and the thiyl radical–forming cysteine is similar, suggesting that allosteric regulation of activity does not involve GRD repositioning. In addition, in contrast to the PcNrdD structural data, we do not observe a dimer-to-tetramer oligomeric state change for StNrdD upon addition of dATP. Instead, both dATP- and ATP-bound states of StNrdD are dimeric, but the positioning of the respective cone domains is vastly different. Together with data from a mutagenesis study and data from hydrogen-deuterium exchange mass spectrometry (HDX-MS), these data suggest that the cone domain position in the presence of dATP leads to an inactive enzyme by anchoring the substrate binding active site flap out of the active site preventing proper substrate positioning in the active site. Structural comparisons of ATP- and dATP-bound states of StNrdD also inform on how nucleotides that differ by one hydroxyl group may lead to the differential cone domain positions. Last, comparisons of StNrdD to the well-studied EcRNR reveal the degree of conservation of allosteric regulation of activity across RNR classes.

## RESULTS

### Structures of StNrdD under ATP- or dATP-binding conditions show markedly different conformations of the cone domain

**Fig. 2.**
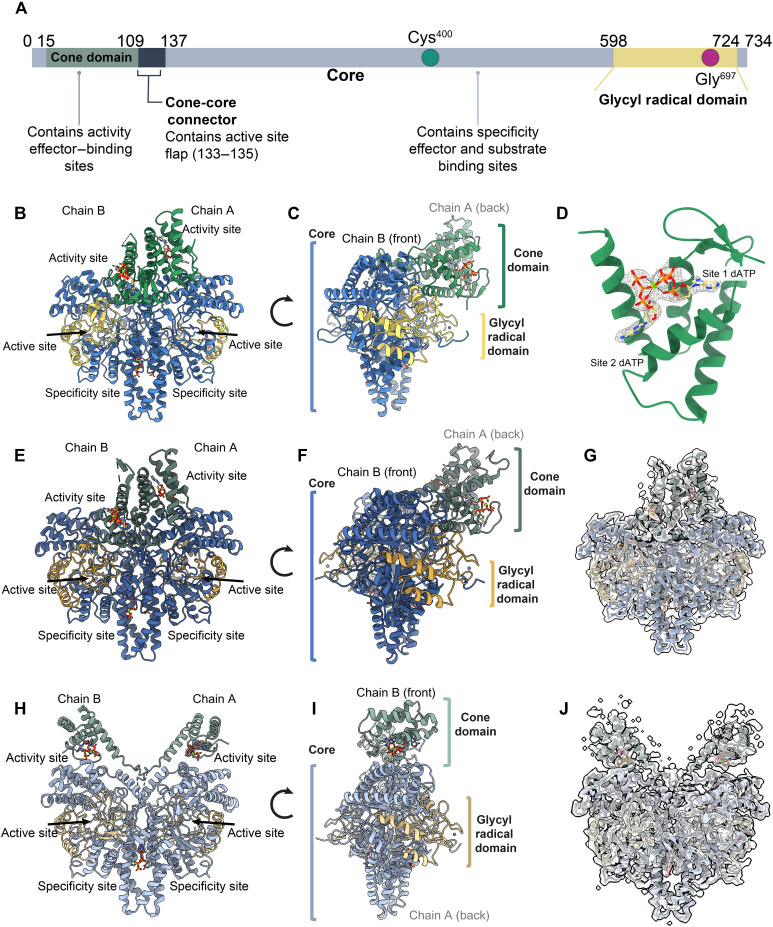
Structures of **StNrdD** with allosteric inhibitor dATP and allosteric activator ATP. (**A**) Domain map of StNrdD with position of thiyl radical Cys^400^ and glycyl radical Gly^697^ indicated. (**B**) Crystal structure (2.6-Å resolution) of dATP-bound (sticks) StNrdD (protein core in blue, GRD in yellow, and cone domain in green). (**C**) Side view of dATP-bound StNrdD crystal structure highlighting the asymmetric center-forward positioning of the cone domains. (**D**) Two molecules of activity effector dATP bound in the cone domain with Mg^2+^ (green sphere). 2*F*_o_-*F*_c_ composite omit density contoured at 1σ for the nucleotides is shown in gray mesh. (**E**) Cryo-EM structure (3.6-Å resolution) of dATP-bound StNrdD (protein core in dark blue, GRD in gold, and cone domains in dark green) resembles crystal structure. (**F**) Side view of dATP-bound StNrdD cryo-EM structure. (**G**) dATP-bound StNrdD structure with cryo-EM map, shown in transparent white volume (contoured at sdLevel 9, step size 1). (**H**) Structure (3.6-Å resolution) of ATP-bound StNrdD (protein core in light blue, GRD in sand, and cone domains in light green). (**I**) Side view of ATP-bound StNrdD. (**J**) ATP-bound StNrdD structure with cryo-EM map, shown in transparent white volume (contoured at sdLevel 9, step size 1).

**Fig. 3.**
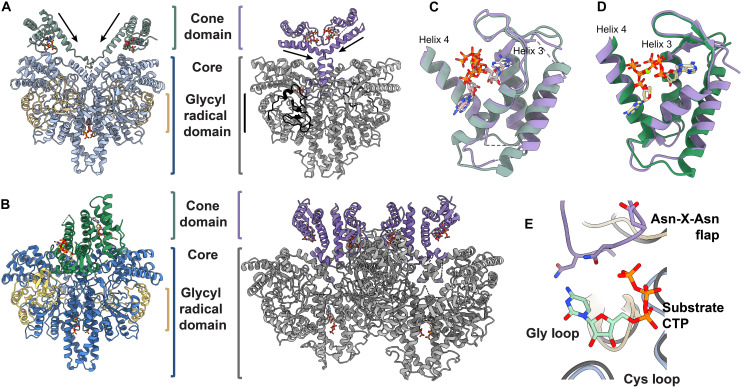
Comparison of the two structures of class III RNRs that have cone domains, StNrdD and PcNrdD. (**A**) Left: ATP-bound StNrdD cryo-EM structure with core domain in light blue, GRD in sand, and cone domains in light green. Right: ATP-bound PcNrdD structure (PDB: 8P23) with core domain in gray, GRD in black, and cone domains in purple. Arrows indicate that the direction of helix 4 of cone domain is pointing toward active site. (**B**) Left: dATP-bound StNrdD crystal structure with core domain in blue, GRD in yellow, and cone domains in green. Right: dATP-bound PcNrdD tetramer (PDB: 8P2C) with core domain in gray, a disordered GRD, and cone domains in purple. (**C**) ATP-bound StNrdD cone domain (ATP carbons in pink and cone domain in light green) overlaid with the ATP-bound PcNrdD cone domain (ATP carbons in white and cone domain in purple). (**D**) dATP-bound StNrdD crystal structure cone domain (dATP in yellow sticks and cone domain in green) overlaid with the dATP-bound PcNrdD cone domain (dATP carbons in white and cone domain in purple). (**E**) Substrate-free ATP-bound StNrdD structure overlaid with the ATP/CTP-PcNrdD structure showing CTP in the active site. Bound substrate (CTP) and ordered active site Asn-X-Asn flap are only seen in the PcNrdD structure.

To investigate the molecular basis of allosteric activity regulation in StNrdD, we used x-ray crystallography and cryo-EM to solve structures of wild-type StNrdD (WT-StNrdD) under nucleotide conditions found to enhance (ATP-bound) or inhibit (dATP-bound) enzyme activity (Fig. 2, tables S1 and S2, and figs. S5 to S8) (*31*). An x-ray structure of StNrdD bound to dATP was determined to 2.6-Å resolution by soaking WT-StNrdD crystals in 10 mM CTP and 10 mM dATP (Fig. 2, B and C). Substrate CTP was not observed in the active site within the 10-stranded α/β barrel, which was expected, given the difficulty in obtaining substrate-bound structures of class III RNRs (*28*). dATP, however, was observed to bind in the cone domains (Fig. 2D) and in the specificity effector sites (fig. S9). The specificity site interactions of effector dATP at the dimer interface are analogous to that observed previously in class III RNR (*17*). Residues Gln^225^ and Glu^293^, which contact the adenine base of the dATP (fig. S9, A and B), are conserved in other class III RNRs (Gln^114^ and Glu^181^, respectively, in T4NrdD) (*17*). Two dATP molecules are bound in the cone domain of StNrdD (Fig. 2D) as was observed recently for PcNrdD (Fig. 3) and discussed in detail below (*28*). Before the PcNrdD structure determination, no more than one dATP has been observed in an RNR cone domain. Two ATP molecules have been observed to bind per cone domain (*22*, *28*, *32*–*36*) but never more than one dATP molecule.

The most unexpected feature of this dATP-bound StNrdD crystal structure is the asymmetric arrangement of the cone domains compared to the dimeric protein core (Fig. 2C). The cone domains, which form an antiparallel dimeric unit, are tilted so they contact only one side of the core fold, the side of the NrdD dimer that contains the opening to the chain B active site within the 10-stranded barrel. The chain B cone domain sits close to the top of the chain B barrel, not only burying a relatively large surface area near the barrel edge (525 Å^2^) but also making contacts with chain A across a chain A–chain B interface (left side of dimer in Fig. 2B). In contrast, the chain A cone domain is on the backside of its 10-stranded barrel, buries less surface area, and only contacts chain A residues (right side of dimer in Fig. 2B). Because of the flexible linker that connects the cone domain to the core, it was possible that crystal lattice contacts were responsible for this asymmetry. Thus, we turned to cryo-EM analysis, which allowed us to obtain StNrdD structures with both ATP and dATP bound in the absence of lattice contacts. We were also curious as to whether any StNrdD tetramers might be observed in the presence of dATP, given that PcNrdD was observed to form tetramers with dATP (*28*).

Cryo-EM samples were prepared using WT-StNrdD incubated with 1 mM TTP specificity effector, 1 mM GTP substrate, and either 3 mM ATP or 3 mM dATP activity effector. These concentrations of ATP and dATP had been shown via liquid chromatography (LC)–MS/MS assays to enhance and inhibit StNrdD enzymatic activity, respectively (*31*). The cryo-EM structure of StNrdD with dATP-bound was solved to 3.6-Å resolution (Fig. 2, E to G, and table S2), with local resolution ranging from ~5.1 Å in the cone domains (residues 15 to 109) to ~3.4 Å in the core (residues 137 to 597 and 725 to 734) (fig. S8). The ATP-bound cryo-EM model was solved to 3.6-Å resolution (Fig. 2, H to J, and table S2), with local resolution ranging from ~5.2 Å in the cone domains to ~3.4 Å in the core (fig. S8). Neither structure had clear density for substrate GTP, whereas both structures have density for the TTP specificity effector bound at the dimer interface (fig. S9, C to F) and both have ordered cone domains with density for two molecules of allosteric activity effectors, dATP or ATP (fig. S10, A to C).

Notably, the cryo-EM structure of StNrdD with dATP is similar to the crystal structure of StNrdD with dATP (Fig. 2, B and E), indicating that lattice contacts are not the source of the cone domain asymmetry. In both cases, two dATP molecules are bound to the cone domains, and the positioning of those two dATP molecules appears to be the same regardless of method used to obtain the structure (fig. S10, A and B). The positioning of the two dATP molecules also resembles that of dATP bound in the PcNrdD structures (Fig. 3D). However, only the dimeric form of StNrdD was seen in any two-dimensional (2D) or 3D classifications across all cryo-EM conditions, suggesting that there is no oligomeric state changes occurring in this enzyme unlike in PcNrdD (Fig. 3, A and B) (*28*).

**Fig. 4.**
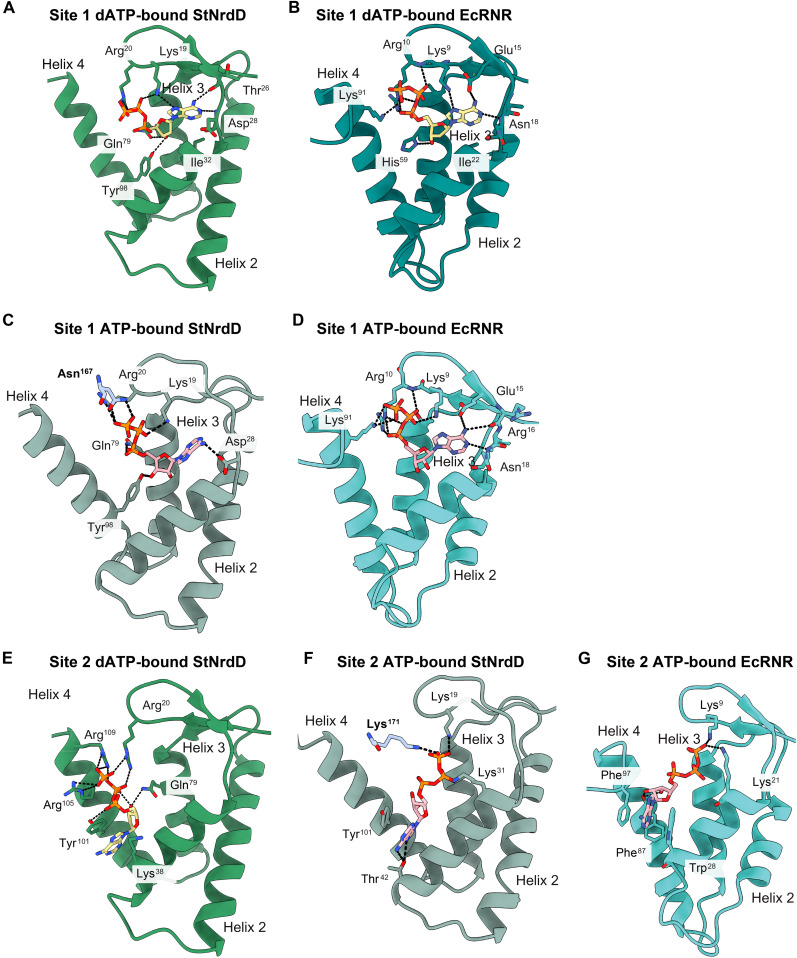
Comparison of ATP and dATP binding in the cone domain between StNrdD structures and RNR structures. (**A**) dATP (carbons are yellow) bound to site 1 in the StNrdD cone domain (green). (**B**) dATP bound to site 1 in EcRNR cone domain (teal) (PDB: 8VHO). (**C**) ATP (carbons are pink) bound to site 1 in StNrdD cone domain (light green). Interactions with core residues (light blue) are in bold labels. (**D**) ATP bound to site 1 in EcRNR cone domain (turquoise) (PDB: 8VHN). (**E**) dATP bound to site 2 in StNrdD cone domain. (**F**) ATP bound to site 2 in StNrdD cone domain. Interactions with core residues are in bold labels. (**G**) ATP bound to site 2 in EcRNR cone domain (PDB: 8VHN). In each panel, dashed black lines represent possible hydrogen bonding interactions with the displayed nucleotide.

The cryo-EM structure of StNrdD with ATP has decidedly different cone domain positions from those of the dATP-bound StNrdD structures (Fig. 2, E, F, H, and I). With two molecules of ATP bound in each cone domain (fig. S10C), the cone domains no longer contact each other and are no longer asymmetric with respect to the protein core (Fig. 2I). The cone domains adopt a conformation along the edges of the dimer, constituting a ~40-Å movement. Such an extensive movement of the cone domains has not been previously identified in any other RNR species. The differences in cone domain positioning in ATP- and dATP-bound states of PcNrdD are also substantial (Fig. 3, A and B), but direct comparison is complicated by the observed change in oligomeric state (*28*). These cone domain differences in the StNrdD structures do not translate to protein core and GRDs (residues 598 to 724), which are relatively similar (Fig. 2, B, E, and H). The cone domains themselves have the same overall four-helix bundle fold as first observed in the prototypical EcRNR (fig. S11A), and despite the large differences in the cone domain positioning in StNrdD, there are no substantial changes in the backbone positions (fig. S11B). There are, however, small but notable shifts at the N-terminal end of helix 3 and the C-terminal end of helix 4 (fig. S11B), as well as differences in the nucleotide positioning and side-chain orientations (Fig. 4). As described below, these differences help explain the molecular basis of the differential cone domain positioning that we observe.

### ATP and dATP appear to bind differently in site 1 of StNrdD’s cone domain due to the presence or absence of a 2′ hydroxyl group

Since there are no apparent differences between dATP binding in the StNrdD crystal structure and the cryo-EM structure and since the crystal structure is of higher resolution, we will use the crystal structure for our dATP-binding structural analyses. We find one dATP molecule bound in the canonical allosteric activity site (site 1) as first identified in EcRNR (Fig. 4, A and B) (*26*). For both StNrdD and EcRNR, the adenine base of the site 1 dATP makes hydrogen bonding interactions with backbone atoms; the phosphates, although positioned somewhat differently, are close to conserved residues from the cone domain hairpin (Lys^9^ and Arg^10^ in EcRNR and Lys^19^ and Arg^20^ in StNrdD), and the 3′ hydroxyl group of the ribose makes a hydrogen bond to a residue of helix 3 (His^59^ in EcRNR and Gln^79^ in StNrdD) (Fig. 4, A and B) (*22*). dATP in EcRNR also forms a hydrogen bonding interaction between the adenine N6 and the side chain of Glu^15^, but that residue is nonpolar in StNrdD. There is an interaction between the terminal phosphate of dATP and Lys^91^ (helix 4) in EcRNR (Fig. 4B) (*22*). StNrdD has one interaction that EcRNR does not have; Tyr^98^ (helix 4) contacts the 3′ OH group of the ribose (Fig. 4A). Tyr^98^ is a Leu in EcRNR.

As shown originally for EcRNR (*26*), ATP also binds to site 1 in StNrdD, but the binding mode is altered from that of dATP (Fig. 4, A and C). It is also altered from that of ATP bound to site 1 in EcRNR (Fig. 4, C and D) (*22*). In EcRNR, ATP and dATP bind similarly to site 1, making the same hydrogen bonding interactions with one important exception; there is a hydrogen bond between His^59^ and the ribose 3′ OH of dATP but not of ATP (Fig. 4, B and D) (*22*). This hydrogen bond loss is critical in EcRNR since it starts a cascade of conformational changes that leads to the creation of a second ATP site (site 2) and to the conversion of the inactive EcRNR state to an active state (*22*). The molecular basis for the hydrogen bond loss for EcRNR is that ATP sits higher in the cone domain to alleviate a close contact between its 2′ OH and Ile^22^ of helix 1 and is thus unable to hydrogen bond to His^59^ (Fig. 4, B and D) (*22*). In StNrdD, ATP also sits higher to alleviate a close contact between its 2′ OH and Ile^32^ of helix 1, the analogous residue to Ile^22^ in EcRNR. This higher positioning of ATP in StNrdD also results in the loss of the hydrogen bond between its 3′ OH and Gln^79^ of helix 3, the analogous residue to His^59^ (Fig. 4, A and C). Instead of Gln^79^ forming a hydrogen bound to the 3′ OH of the dATP ribose (fig. S10E), Gln^79^ is disordered in the ATP structure. However, unlike EcRNR, the loss of the 3′ OH hydrogen bond is not the only difference between dATP and ATP binding.

**Fig. 5.**
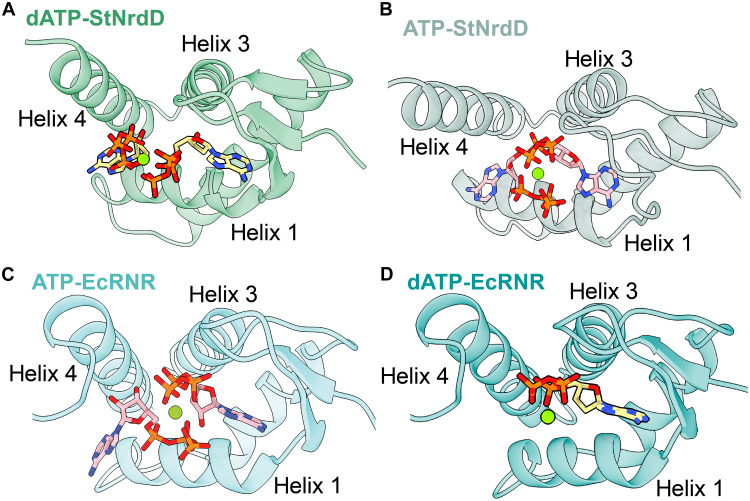
Top-down comparison of nucleotide binding in the cone domains of StNrdD and EcRNR. (**A**) Top-down view of dATP-StNrdD cone domain (transparent green; dATP carbons in yellow opaque sticks). (**B**) Top-down view of ATP-StNrdD cone domain (transparent light green; ATP carbons in pink opaque sticks) (PDB: 8VHN). (**C**) Top-down view of ATP-EcRNR cone domain (transparent turquoise; ATP carbons in pink opaque sticks) (PDB: 8VHN). (**D**) Top-down view of dATP-EcRNR cone domain (transparent teal; dATP carbons in yellow opaque sticks) (PDB: 8VHO). Green sphere represents Mg^2+^.

The adenine base of ATP bound to site 1 in the cone domain of StNrdD fits better in the cryo-EM map with a ~60° rotation from that observed for the adenine base of dATP (fig. S10D). This rotation alters the adenine ring hydrogen bonding from that of dATP-bound StNrdD (Fig. 4, A and C) and also from that of ATP-bound EcRNR (Fig. 4, C and D). A more important difference than the rotation of the base is the observation that ATP binds in an extended conformation compared to dATP (Figs. 4, A and C, and 5, A and B; and fig. S10, A to C). The variation in conformation between dATP and ATP is evident, despite the modest resolution of the maps in part due to the strong density features associated with the three phosphate moieties (fig. S10, A to C). The more extended conformation of ATP in site 1 is made possible by the loss of the helix 3 Gln^79^-nucleotide interaction and N terminus of helix 3 shifting outward, expanding this nucleotide-binding site. With the increased space, the terminal phosphate of ATP extends ~5.5 Å farther from the adenine base than it does in the case for dATP (Fig. 5, A and B). Possible contacts to the phosphates of ATP in their extended positions include cone domain hairpin residues Lys^19^ and Arg^20^ and, notably, core domain Asn^167^ (Fig. 4C). To the best of our knowledge, this Asn^167^-ATP interaction is the first contact observed between a cone domain nucleotide and a non–cone domain amino acid residue.

### ATP and dATP bind differently in site 2 of StNrdD’s cone domain due to differential binding of the nucleotide in site 1

The second nucleotide-binding site in the cone domain is referred to as site 2. Here, the adenine base of ATP or dATP is positioned between helix 1 and helix 4 (Fig. 4, E to G). Although the adenine location is consistent between dATP and ATP in StNrdD and ATP in EcRNR, the phosphate positions are distinct (Fig. 4, E to G). The phosphates of dATP run up along helix 4, making contacts with helix 4 residues Tyr^101^ (Phe^97^ in EcRNR), Arg^105^ (Lys^91^ in EcRNR), and Arg^109^ (Fig. 4E), whereas the phosphates of ATP in both EcRNR and StNrdD stretch across the cone domain contacting helix 1 residue Lys^21/31^ and hairpin residue Lys^9/19^ (Fig. 4, F and G). For StNrdD, there is also a contact to core domain residue Lys^171^ (Fig. 4F). In all cases, the phosphate moieties of both nucleotides coordinate a shared Mg^2+^ ion (Fig. 5, A to C).

**Fig. 6.**
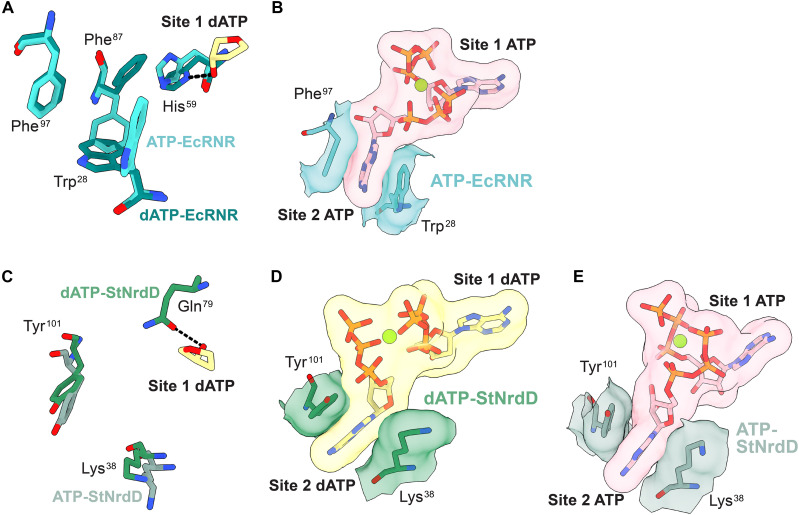
Cone domain of EcRNR undergoes a conformational change when ATP binds that creates a second nucleotide-binding site, whereas the second nucleotide-binding site in StNrdD is always present. (**A**) In EcRNR, His^59^, Phe^87^, and Trp^28^ undergo conformational changes to accommodate two molecules of ATP (dATP only binds in site 1). Conformation of residues in ATP-EcRNR structure (PDB: 8VHN) are shown in turquoise, whereas the conformation of residues in dATP-EcRNR structure (PDB: 8VHO) are shown in teal. Ribose from dATP is shown in yellow. The remainder of dATP is not shown for clarity. ATP is not shown. (**B**) Space filling model of the packing of the ATP molecules (pink) between helix 4 residue Phe^97^ and helix 1 residue Trp^28^ in ATP-EcRNR (turquoise). (**C**) In StNrdD, the hydrogen bond to the ribose of dATP and Gln^79^ is lost when ATP binds. Gln^79^ is disordered when ATP is bound. ATP-StNrdD residues are shown in light green. dATP-StNrdD residues are shown in green with ribose of dATP in yellow. Remainder of dATP is not shown for clarity. (**D**) Space filling model of the packing of one of the two dATP molecules (yellow) between helix 4 residue Tyr^101^ and helix 1 residue Lys^38^ in dATP-StNrdD (green). (**E**) Space filling model of the packing of one of the two ATP molecules (pink) between helix 4 residue Tyr^101^ and helix 1 residue Lys^38^ in ATP-StNrdD (light green).

EcRNR site 2 does not exist when dATP is bound in site 1 (Fig. 5D) and His^59^ is positioned to hydrogen bond to the 3′ OH group of dATP (Fig. 6A). In contrast, with ATP bound in site 1 in EcRNR, the hydrogen bond to the 3′ OH and helix 3 residue His^59^ is lost, and His^59^ moves. This side-chain movement flips the position of the side chain of Phe^87^ (helix 4), which, in turn, moves the side chain of Trp^28^ (helix 1) (Fig. 6A). The movement of the side chain of Phe^87^ (helix 4) creates space for the ribose of the second nucleotide in EcRNR, and the movement of the side chain of Trp^28^ (helix 2) creates space for the adenine of the second nucleotide in EcRNR (Fig. 6B). In their new positions, Phe^87^ and Trp^28^, along with Phe^97^ (loop following helix 4), form the binding pocket for the second ATP (Fig. 6B). In StNrdD, no rearrangement is needed to bind a second nucleotide molecule, as a binding pocket is preformed with Tyr^101^ (helix 4) and Lys^38^ (helix 1) already positioned to stack against the adenine base (Fig. 6, C to E).

**Fig. 7.**
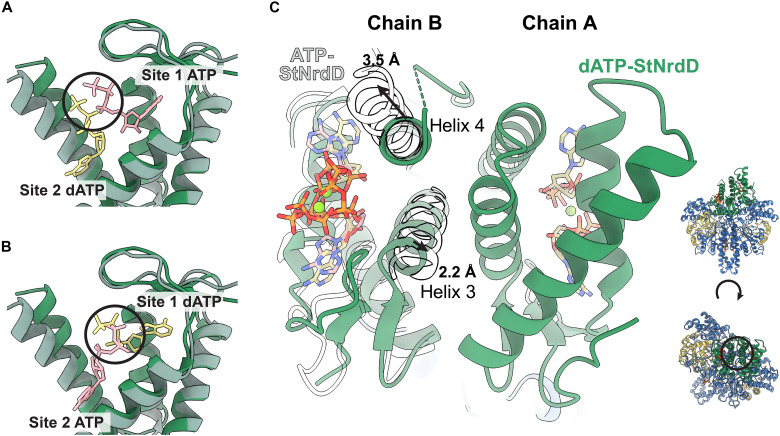
The differential binding of ATP and dATP to the StNrdD cone domain and the effect on the orientation of cone domain helices. (**A**) ATP-bound cone domain (ATP in pink and cone domain in light green) superimposed with dATP-bound cone domain (dATP in yellow and cone domain in darker green). Site 2 ATP and Site 1 dATP are not shown for clarity. Circle highlights the unfavorably close interactions between phosphates that would be made if ATP bound similar to dATP in site 2. (**B**) ATP-bound cone domain (ATP in pink and cone domain in light green) superimposed with dATP-bound cone domain (dATP in yellow and cone domain in darker green). Site 1 ATP and site 2 dATP are not shown for clarity. Circle highlights the unfavorably close interactions between phosphates that would be made if dATP bound similar to ATP in site 2. (**C**) Expansion of cone domain by ATP binding explains the breaking of the cone domain dimer. Helix 3 of one cone domain moves unfavorably close to helix 3 of the other cone domain, and helix 4 of one cone domain moves away from the helix 4 of the other cone domain, resulting in the loss of favorable interactions. One ATP-bound StNrdD cone domain (transparent, outlined in black) is aligned to chain B of the dATP-bound StNrdD. Helices 3 and 4 are labeled, and the distances that the helices move between the ATP- to dATP-bound states are labeled. The viewing angle is indicated by the black circle on the structure inset on the bottom right of the panel (dATP-bound StNrdD structure shown with the protein core in blue, GRD in yellow, and cones in green).

Given that nucleotide-binding site 2 in StNrdD is preformed, the question arises as to why ATP and dATP bind differently to this site. The answer appears to be simple. The extended conformation of ATP in site 1 blocks the phosphate-binding site used by dATP in site 2 (Fig. 7A). Likewise, a second dATP cannot bind similarly as the second ATP because the phosphates of dATP in site 1 are already occupying this binding site (Fig. 7B). That is, to fit two nucleotides into a small four-helix bundle, the first puzzle piece restricts the positioning of the second. In this case, the fit of the first puzzle piece depends on the presence or absence of a 2′ OH group on the nucleotide, and then the fit of the second piece depends on the fit of the first. Two ATP molecules fit when they both adopt extended conformations (Fig. 5, B and C), and two dATP molecules fit when they are both in compact conformations (Fig. 5A). Notably, these nucleotide binding modes are similar to the nucleotide binding modes observed in PcNrdD (Fig. 3, C and D).

### ATP binding to the cone domains cause helices 3 and 4 to shift and that shift is incompatible with the dimeric arrangement of cone domains that is observed in the dATP structure

As described above, the structure of StNrdD in the inactive state shows both cone domains interacting as a dimeric unit and tilted over the core protein fold (Fig. 2E). In contrast, the active structure shows the cone domains separated (Fig. 2H). Structural comparisons allow us to postulate as to how ATP binding causes the dimeric cone domain unit to come apart, leading to the active conformation of the enzyme. When ATP displaces dATP in a cone domain, both helices 3 and 4 shift. Helix 3′s small outward shift in the presence of ATP (~2.2-Å tilt) (Fig. 7C) appears to be due, at least in part, to the loss of the only hydrogen bonding contact to the site 1 nucleotide (Gln^79^ to 3′ OH of dATP) when ATP binds (Figs. 4, A and C, and 6C). Helix 4’s larger shift (~3.5-Å tilt) (Fig. 7C) appears to be due to the loss of multiple interactions of helix 4 residues when ATP displaces dATP in site 2 (Fig. 4, E and F). That is, dATP binding appears to anchor helices 3 and 4 within a compact cone domain, whereas ATP binding appears to free helices 3 and 4, expanding the cone domain. These movements are important because of the arrangement of helices 3 and 4 within the dimeric cone domain unit: Helix 3 of one cone domain packs against the helix 3 of the other cone domain in an antiparallel configuration, and helix 4 of one cone packs against helix 4 of the other cone, again in an antiparallel configuration (Fig. 7C and fig. S12). Outward tilting of helix 3 would lead to unfavorably close interactions between the helix 3 helices, whereas outward tilting of helix 4 would break favorable packing interactions between the helix 4 helices (Fig. 7C). With the generation of an unfavorable interaction between one set of the helix pairs and the loss of the favorable interaction between the other helix pair, the dimer interface should be broken. With the interface broken, the cone domains would be expected to move apart (Fig. 7C). We find the cone domains resting against the protein core (Fig. 2H), stabilized in part by the favorable electrostatic interactions made by core residues Lys^171^ and Asn^167^ to ATP phosphates (Fig. 4, C and F). Unfortunately, mutations designed to break the dimer interface and confirm the importance of cone domain dimerization to allosteric inhibition have led to insoluble protein thus far. Studies are ongoing.

### Structural analysis shows that dimerization of the cone domains due to dATP binding restricts movement of residues of a connector region that include the active site flap

**Fig. 8.**
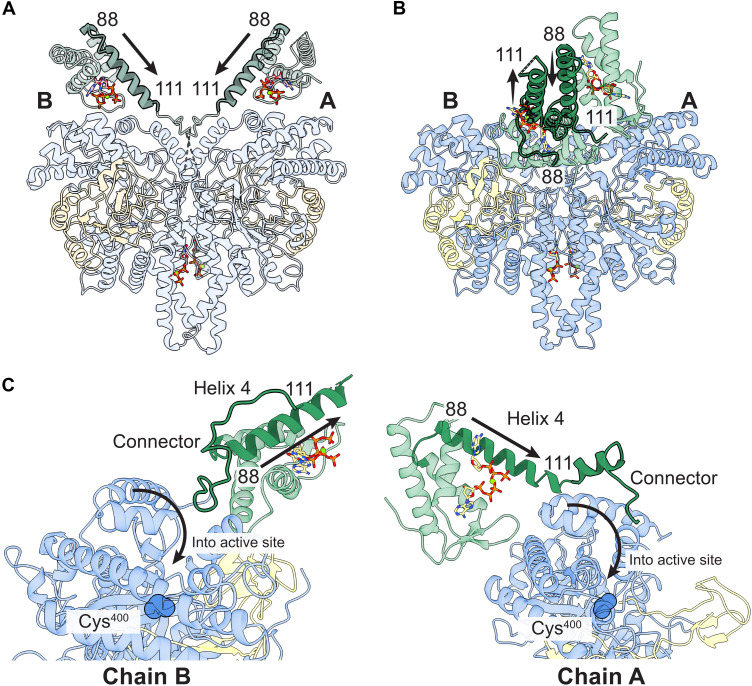
Cone domain position affects availability of active site flap to facilitate catalysis. (**A**) With ATP bound to the cone domains, cone domain positions orient helix 4 (residues 88 to 111) and thus the connector (residues 111 to 137) into the active sites so that the active site flap (residues 133 to 135) can be positioned properly for catalysis. Residues 88 and 111 are labeled. The core of ATP-StNrdD is in transparent light blue, GRD is in transparent sand, and cone domains are in transparent light green with helix 4 in opaque light green with black outline. (**B**) With dATP bound to the cone domains, the connector (residues 111 to 137) and the active site flap (residues 133 to 135) are unavailable for catalysis. Chain B helix 4 (residues 88 to 111) is pointing away from the chain B active site barrel, and although chain A helix 4 is pointing toward the chain A active site barrel, the chain A cone domain is on the opposite side from the chain A barrel opening. The core of dATP-StNrdD is in transparent blue, GRD is in transparent yellow, and cone domains are in transparent green with helix 4 in opaque green with black outline. (**C**) Left: Close-up view of chain B showing helix 4 (residues 88 to 111) pointing away from the chain B active site (catalytic Cys^400^ shown in spheres). The connector (residues 111 to 137), which is outlined in black, is restrained by the cone domain from reaching the chain B active site. Right: Close-up view of chain A showing that although helix 4 (residues 88 to 111) is pointed toward the active site (catalytic Cys^400^ shown in spheres), the partially ordered connector cannot reach the active site. Entry into the active site is indicated with a black arrow.

The cone domains are connected to the core domains by a connector region (residues 111 to 137 in StNrdD). This connector region contains the previously identified active site flap with the sequence motif of Asn-X-Asn (*28*). The PcNrdD structure showed that one Asn of the motif is close enough to hydrogen bond to the substrate (Fig. 3E) and the other Asn contacts the substrate specificity loop and the GRD. The Asn-X-Asn sequence motif is conserved in NrdD and is composed of residues 133-Asn-Ala-Asn-135 in StNrdD (fig. S13). In all StNrdD structures, the active site barrels are open; no active site has substrate or has a closed active site flap (fig. S14). The connector region does not have clear density to accurately build the full-length connector in any of the StNrdD structures. The dATP-StNrdD crystal structure has the most intact connector. Chain A is only missing residues 132 to 135, and chain B is only missing residues 109 to 115. Fortunately, these structural data are sufficient to rationalize why the observed positions of the dATP–cone domains generate an inactive NrdD. We can also rationalize why the observed positions of the ATP–cone domains generate an active NrdD, despite the fact that the ATP-bound StNrdD cryo-EM structure is missing most of the connector region (residues 120 to 137 are disordered). First, an active structure requires that the 133-Asn-Ala-Asn-135 flap can reach into the active site barrel to contact substrate. For this movement to happen, the C terminus of helix 4 of the cone domain must be oriented toward the active site barrel so that the connector (residues 110 to 137) is pointed in the right direction. In addition, the connector region must be unconstrained, such that the residues can descend into the barrel. These criteria are met in the ATP StNrdD structure as helix 4 of each cone domain points toward the respective active site barrel (Fig. 8A), and connector residues are unrestrained, so much so that these residues are disordered in the structure.

In contrast, these criteria are not met when dATP is bound in the cone domains (Fig. 8B). We find that the chain B cone domain is near the opening of the chain B barrel but the cone domain is rotated, such that the C terminus end of helix 4 is pointing away from the barrel opening (~180° away), pulling the connector in the opposite direction from the active site. The connector residues are also restrained in this remote location, which is likely why they appear ordered in the dATP structure. The first part of the chain B connector (residues 119 to 127) runs along the cone domain dimer interface composed of the two helix 4 helices, making several backbone–side chain hydrogen bonding contacts. The second part of the connector (residues 128 to 132) runs along a loop (residues 43 to 47) that connects chain B cone domain helix 1 to helix 2, making one hydrogen bond. The last part of the connector (residues 133 to 138) contacts chain B cone domain helix 2 (residues 49 to 57) (Fig. 8C, left), making an additional hydrogen bond. That is, the chain B connector is wrapped around the chain B cone domain similar to a thread around a spool, making it impossible for the Asn-X-Asn active site flap to sequester substrate in the active site for catalysis.

The dATP-bound cone domain in chain A is a different story. Here, the C terminus of the cone domain helix 4 is pointing in the correct direction toward the chain A active site, but the chain A cone domain is sitting on the opposite side of the NrdD dimer from the barrel opening and is thus too far away for the connector to reach the active site (Fig. 8C, right). The asymmetry of the dATP-bound structure is therefore part of the mechanism of inhibition. One connector with its active site flap is prevented from reaching into the active site by wrapping its cone domain, whereas the other is kept out of its active site by wrapping its cone domain around the side of the protein core.

### Levels of HDX protection differ between ATP- and dATP-bound effector conditions and are consistent with the observed cone domain dimer in the dATP state and with an active site flap that can close in the ATP state

**Fig. 9.**
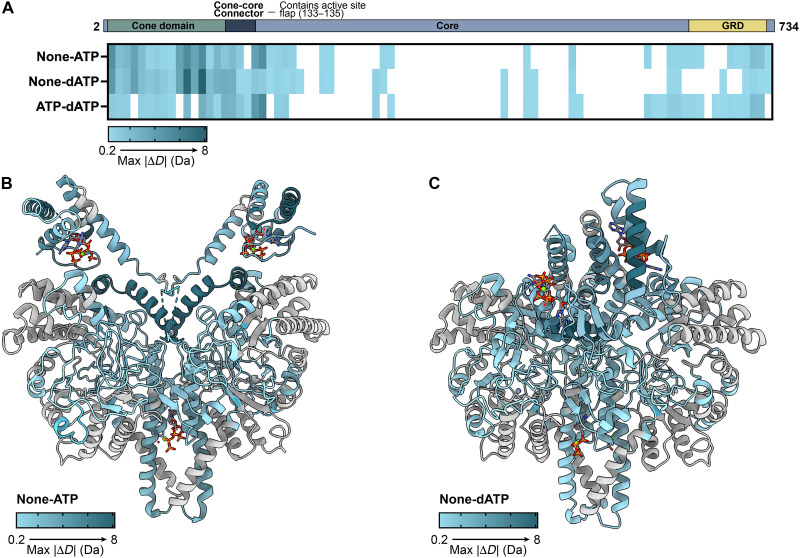
Regions showing greatest differences in HDX in StNrdD in the presence or absence of allosteric activity effectors ATP and dATP. HDX experiments using WT-StNrdD were performed under ATP-binding (1 mM GTP, 1 mM TTP, and 3 mM ATP), dATP-binding (1 mM GTP, 1 mM TTP, and 3 mM dATP), and no activity effector (“none,” 1 mM GTP, and 1 mM TTP) conditions. Exchange reactions were incubated for 20 s, 1 min, 10 min, 1 hour, or 2 hours before quenching and freezing. (**A**) HDX differences between the no activity effector and ATP-binding condition shown linearly from N terminus to C terminus of the primary structure. Each row shows a pairwise comparison of the maximum absolute difference in deuterium uptake (|Δ*D*|, in daltons) of peptides with statistically significant [98% confidence interval (CI)] differences from each listed condition. Peptides that are below the 98% CI difference are white. Shade darkens with the value of |Δ*D*| for a given peptide, indicating a notable difference between the states in that region. (**B**) HDX differences between the no activity effector and ATP-binding condition are mapped onto the StNrdD-ATP cryo-EM structure. Shade darkens with the value of max |Δ*D*| (in daltons), corresponding to part (A). Gray color indicates that there was no differences in exchange above the 98% CI. (**C**) HDX differences between the no activity effector and dATP-binding condition are mapped onto the StNrdD-dATP crystal structure. Shade darkens with the value of max |Δ*D*| (in daltons), corresponding to part (A). Gray color indicates that there was no differences in exchange above the 98% CI. Bound nucleotides are in sticks.

HDX-MS was pursued to obtain solution state data to complement the StNrdD structures. The residues that experience greatest differences in HDX properties upon ATP or dATP binding are in the cone domains (Fig. 9A). Mapping regions with significant HDX differences between ATP- and dATP-bound conditions onto the respective StNrdD structures for each state, we find that addition of ATP protects structural regions outlining the active site pocket (Fig. 9B). This result is consistent with the above proposal that in the presence of activity effector ATP, the connector region containing the active site flap can close over and protect the active site. In contrast, dATP binding induces protection in helices along the cone-cone dimer interface, supporting the relevance of the structurally observed dATP-bound cone domain dimer (Fig. 9C).

Given the inherent flexibility of the connector region, we wondered whether the cone domains can assume different orientations with respect to the protein core than what we observe in these structural snapshots. In theory, multiple orientations of the dATP-bound cone domain dimer with respect to the protein core should be able to restrict the movement of active site flap residues to the active site barrel. In addition, it should be possible for multiple positions of the ATP-bound cone monomers to allow the active site flap residues to access the active site. Although we do see differences in HDX in core residues when ATP and dATP are added, we cannot say that the orientation of the cone domains observed here is exclusive.

### Truncations of the cone domain and connector region of StNrdD (Δ1 to 137) abolish substrate turnover, while addition back of the connector region (Δ1 to 115) restores some of the lost enzyme activity

To gain additional support for the proposal that the cone domain (residues 1 to 111) and/or connector region (residues 111 to 137) play a role in substrate binding/catalysis, we carried out activity assays using StNrdD variants. Two StNrdD variants were prepared: a variant missing the cone domain only (ΔConeOnly-StNrdD, residues 1 to 115 deleted) and a variant missing both the cone-core connector and the cone domain (ΔCone-ΔConnector-StNrdD, residues 1 to 137 deleted). These variants were assayed in the presence of 1 mM TTP as the specificity effector, 1 mM GTP as the substrate, and 3 mM ATP or dATP as the activity effector, or no activity effector. To ensure that these truncations were not negatively affecting glycyl radical installation or overall protein stability, we characterized the radical content of each variant with electron paramagnetic resonance (EPR) spectroscopy and assessed the secondary and tertiary structure of each variant by circular dichroism (CD). No substantial difference in protein folding (fig. S15) or radical content (fig. S16) was observed for the truncated variants relative to the WT enzyme. Following installation and quantification of glycyl radical, each of the StNrdD variants was incubated with the different nucleotide conditions, and the total concentration of product, dGTP, was measured. WT-StNrdD produced 397 ± 34 pmol of dGTP in the presence of activity effector ATP and specificity effector TTP (fig. S17). WT-StNrdD produced 53.2 ± 13 pmol of dGTP in the presence of the specificity effector TTP alone (fig. S17). When dATP is used as the activity effector, WT-StNrdD produced only 1.07 ± 0.36 pmol of dGTP, consistent with dATP serving as an allosteric enzyme inhibitor (fig. S17). Removal of both the cone domain and cone-core connector (ΔCone-ΔConnector-StNrdD) results in undetectable product turnover under all activity effector conditions studied (fig. S17). However, when the cone domain alone is truncated (ΔConeOnly-StNrdD), GTP turnover proceeds in the absence of allosteric inhibitor dATP, although at a lower yield than that observed for the WT-StNrdD (fig. S17). These data are consistent with the proposal that the connector region is essential for catalysis. GTP turnover for the ΔConeOnly-StNrdD variant is lower when ATP or dATP is present compared to the TTP/GTP-alone condition. This decrease in activity is likely due to the competitive binding of ATP and dATP with GTP and TTP for the active site and specificity sites, respectively.

### The GRD is ordered in both ATP- and dATP-bound structures of StNrdD

Before these structural studies, we postulated that the cone domains could regulate RNR activity by regulating the position of the GRD and therefore radical transfer to the catalytic cysteine. In contrast to what was observed in the PcNrdD cryo-EM structures (*28*), we do not find a correlation between dATP binding in the cone domain and the disordering of the GRD. The GRD is ordered in both of our ATP and dATP structures (Fig. 2). Since a distance increase between the glycyl radical on the Gly loop and the catalytic cysteine on the Cys loop could inhibit enzyme activity, we compared the Gly loop to Cys loop interactions in all structures. No meaningful differences in loop interactions were observed between ATP- and dATP-bound structures (fig. S18, A to D). In addition, the EPR spectra are the same regardless of which activity effector is bound (fig. S19), indicating that environment of the glycyl radical does not change.

## DISCUSSION

We carried out these studies to probe the structural basis of allosteric activity regulation in a class III RNR. In starting this work, we considered two possible mechanisms for how dATP binding to the regulatory cone domain could inactivate a class III RNR enzyme: inactivation by preventing radical transfer from the glycyl radical species to the catalytic cysteine and inactivation by preventing the proper binding of the substrate for radical-based catalysis. Class Ia RNRs regulate activity by preventing radical transfer when dATP levels become high (*21*), and recent studies of PcNrdD have suggested that both regulatory mechanisms may be at play for class III RNR (*28*).

**Fig. 10.**
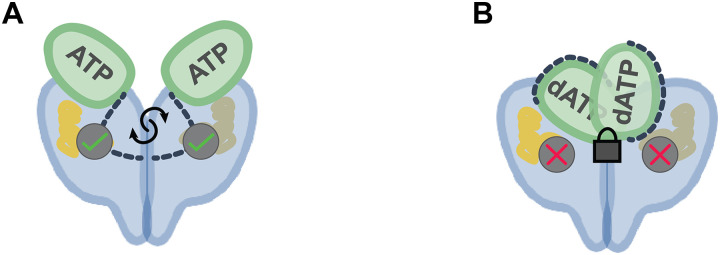
Proposed model of activity regulation in class III RNRs. (**A**) The connector region with the active site flap (dashed lines) is free to enter the active site, allowing for catalysis, when ATP is bound in the cone domains. (**B**) The connector region with the active site flap (dashed lines) is restrained by the cone domains and is unable to enter the active site when dATP is bound in the cone domains.

Our current data support only the second proposal that allosteric regulation in a class III RNR involves regulation of substrate binding (Fig. 10). The Asn-X-Asn active site flap, which is contained within the connector region (residues 111 to 137), is restricted from moving into the active site to bind substrate when dATP is bound in the cone domains. Our truncation study confirms that the connector region is essential for catalysis in StNrdD. In contrast, ATP binding repositions the cone domain, such that the connector region is freely available for substrate binding. This allosteric regulatory mechanism is a departure from that of EcRNR, but there is an important similarity. In both cases, the dATP inhibitory mechanisms involve the restriction of amino acid residue movement into the active site. In EcRNR, it is the movement of β_2_ residues into the active site that is restricted by dATP binding to the cone domain, and in StNrdD, it is the movement of connector region residues that is restricted.

The molecular mechanisms by which the cone domains restrict residue movement also show parallels between EcRNR and StNrdD. Interfaces formed between the α_2_ cone domains and β_2_ in EcRNR restrict β_2_ movement by sequestering it within an α_4_β_4_ ring structure (fig. S4A) (*22*). β_2_ cannot move when it is in the ring. The β-α interfaces that stabilize the ring form only when dATP is bound to the cone domain. dATP binding causes a contraction of the cone domain, which, in turn, causes one turn of a cone domain helix to unwind, creating a binding surface on α for β (*22*). It similarly appears that dATP binding to StNrdD causes a cone domain contraction, but instead of creating α-β interfaces, cone domain contraction in StNrdD causes dimerization. This cone domain dimerization appears to restrain movement of the connector region. Thus, in both cases, dATP binding creates a protein-binding surface that restrains another component of the protein from adopting its active conformation. RNR is apparently using a molecular version of the children’s game “keep-away.” In addition, for both *E. coli* (*22*) and *S. thermophilus* RNRs, ATP binding results in an expansion of the cone domain (figs. S4B and 7C), which breaks the protein-protein interfaces, freeing the respective protein components to adopt active conformations.

The next question we considered is whether other class III RNRs will use the same allosteric mechanism as StNrdD. Here, the recent PcNrdD cryo-EM structures (*28*) allow for comparison. In PcNrdD, as in StNrdD, the cone domain positions also differ depending on whether ATP or dATP is bound, but the positions adopted by the cone domains in PcNrdD are not the same as in StNrdD (Fig. 3, A and B). In particular, the ATP-bound PcNrdD cone domains do not contact the protein core as they do in StNrdD and are instead pointing directly up (Fig. 3A). The dATP-bound PcNrdD cone domains form a dimer that is distinct from StNrdD. It is antiparallel, whereas the StNrdD cone domain dimer is parallel. In addition, the presence of dATP causes the PcNrdD core to tetramerize (Fig. 3B). However, from a mechanistic perspective, the ATP-bound PcNrdD structure is analogous to the ATP-bound StNrdD structure, and the dATP-bound structures are analogous to each other. In both class III RNRs, ATP-bound cones are oriented, such that the connector region is directed toward the active site barrel (Fig. 3A), allowing the Asn-X-Asn active site flap to enter the active site (Fig. 3E), and in both class III RNRs, dATP-bound cone domain positioning holds the connector region out of the active site. Thus, despite the structural differences, the same allosteric mechanism can be invoked: dATP inhibits enzyme activity by restraining the connector region, and ATP promotes enzyme activity by freeing the connector region (Fig. 10). A next step for PcNrdD is to confirm the relevance of the tetrameric species to allosteric inactivity by dATP. Our prediction is that the tetramer will be relevant and that tetramerization will assist in holding the connector region out of the active site.

On the basis of the above analysis, it does appear that both PcNrdD and StNrdD use regulation of substrate positioning in their allosteric activity mechanisms, but the question arises as to whether PcNrdD also uses regulation of radical transfer as part of its mechanism. In the PcNrdD structures with dATP, the GRDs were consistently observed to be disordered and Bimaï *et al.* (*28*) proposed that GRD disordering is part of the molecular mechanism of allosteric activity regulation. As mentioned above, our structural data do not support this proposal, as the GRDs in our dATP-bound structures are ordered and are within the active site barrel. We find no notable difference in the orientations of the Gly loop and Cys loop when comparing dATP-bound to ATP-bound structures, nor do we find notable differences in the glycyl radical EPR spectra to suggest a rearrangement of the Gly loop or GRD in response to dATP binding. Bimaï *et al.* (*28*) also state that the EPR signal for the glycyl radical is the same in ATP- and dATP-bound states of PcNrdD, again suggesting that the radical environment is similar between the two conditions. We suspect that the observed disorder of the GRD in the dATP-bound PcNrdD structures is due to the inherent flexibility of the GRD in enzyme that has not been activated, flexibility that is required for interaction with NrdG.

Despite the new wealth of structural data on NrdD enzymes, it remains an open question as to whether all cone domain–containing class III RNR enzymes will share a common allosteric activity mechanism. Work on class Ia RNRs has shown that whereas some species use an almost identical molecular mechanism for allosteric regulation, others use variations on the same theme. In all class Ia RNR enzymes investigated to date, the allosteric activity mechanism involves keeping β_2_ away from α_2_, but the molecular method used to keep β_2_ away is not the same (*21*, *25*, *34*, *36*–*41*). For example, *E. coli* (*21*), *Neisseria gonorrhoeae* (*37*), *Clostridium botulinum* (*38*), and *Aquifex aeolicus* (*34*) class Ia RNRs share the use of an α_4_β_4_ ring to keep β_2_ subunit at arm’s length from α_2_, but human class Ia RNR uses oligomerization of α, at least in part, to prevent β_2_ from binding productively for radical transfer (*25*, *39*, *40*). The differences in allosteric regulation between RNRs, even RNRs of the same subclass, encourages the pursuit of RNRs as antibiotic targets. Targeting class III RNRs could be especially helpful in the gut microbiome, where the threat of antibiotic resistance looms and where large fractions of obligate and facultative anaerobes contribute to the spread of antibiotic resistance genes to other bacteria through horizontal gene transfer (*42*, *43*).

Beyond RNR, the targeting of allosteric sites on enzymes is an attractive alternative to use of mechanism-based or competitive enzyme inhibitors. All too often, however, details of allosteric regulation remain enigmatic hindering drug design efforts. In this regard, studies of RNR can provide insight, revealing often-unexpected modes of allosteric regulation that may be relevant beyond RNRs. There is more than one way to down-regulate enzyme activity, and structural studies of RNRs are revealing a wide variety of nature’s solutions to this challenge.

In addition to RNR allosteric activity regulation having been enigmatic, there is a lot that we do not know about allosteric substrate specificity regulation. Obtaining RNR structures with substrates bound has been a challenge for all RNR enzymes including class III, which was first structurally characterized in 1999 (*44*). Although we added both GTP substrate and TTP specificity effector to our cryo-EM solutions, we do not see clear density for GTP. The nucleotide conditions we used in the cryo-EM were also used in enzyme assays that displayed product turnover (*31*). One hypothesis for this apparent discrepancy between the biochemical and structural data is that substrate might bind in StNrdD more tightly when glycyl radical is present. Protein used in LC-MS/MS assays and for EPR spectroscopy is activated, but protein used in the cryo-EM experiments is not. Obtaining additional structures of class III RNRs with substrates and specificity effectors remains a high priority.

In summary, this structural work in addition to the snapshots obtained by Logan and co-workers of PcNrdD (*28*) have markedly advanced our understanding of class III RNRs. However, the work is not complete. We predict that class III RNRs will continue to fascinate and frustrate researchers for some time to come. RNRs are amazing enzymes that hold their secrets tightly, requiring extensive structural and biochemical studies to unveil their molecular mechanisms. However, as the only de novo source of deoxyribonucleotides in a cell, RNRs deserve the attention that they command. The applications of this research for drug development are considerable.

## MATERIALS AND METHODS

### Materials

All chemicals, solvents, and reagents were purchased from Sigma-Aldrich unless otherwise noted. The sodium chloride for buffer components was purchased from Thermo Fisher Scientific. AdoMet was gifted by the Bandarian laboratory.

### Protein cloning, expression, and purification

StNrdD, originally amplified from a commercial yogurt sample, was cloned into a pET15(b) vector as N-terminally His_6_-tagged protein with a thrombin cleavage sequence. All StNrdD variants were made via site-directed mutagenesis using the Q5 Site-Directed Mutagenesis Kit (New England Biolabs) using the primers summarized in table S3. Sanger sequence verification was performed by Quintara Biosciences.

All StNrdD proteins were expressed in T7 Express cells (New England Biolabs) and purified as previously described (*31*). *S. thermophilus* NrdG (StNrdG) was expressed from a pET28(a) vector as a codon optimized His_6_-tagged protein with a thrombin cleavage site (codon optimized sequence WP_011681737.1 synthesized by GenScript) in T7 Express cells (New England Biolabs). StNrdG was expressed, purified, and reconstituted as previously described (*31*), with the following changes. Following immobilized metal affinity chromatography via TALON column (Cytiva) and subsequent buffer exchange, protein concentration was measured via absorbance at 280 nm (*A*_280_) before sealing in a bottle and the headspace degassed by sparging with argon gas. Then protein was brought into an anaerobic chamber with a 100% nitrogen atmosphere (MBRAUN) maintained at 4°C, opened, and dithiothreitol was added to 5 mM concentration. Protein sat vented for at least 24 hours in the MBRAUN at 4°C before reconstitution. Reconstitution was performed as previously described (*31*), yielding ~30% recovery of protein at ~3.1 Fe/monomer as measured by a ferene assay, as previously described (*37*), with protein quantified by *A*_280_ using extinction coefficients: 93,630 M^−1^ cm^−1^ for WT-StNrdD, 86,180 M^−1^ cm^−1^ for ΔCone-ΔConnector-StNrdD, and 86,180 M^−1^ cm^−1^ for ΔConeOnly-StNrdD, and Bradford protein assay with a correction of 0.69. Reconstituted protein was stored in a 4°C refrigerator inside an anaerobic MBRAUN chamber.

### Circular dichroism

Each sample consisted of protein (0.3 mg/ml) in 50 mM sodium phosphate (pH 7.6). Samples (250 μl) were loaded into 1-mm pathlength rectangular quartz cuvette (JASCO) and analyzed using a JASCO J-1500 CD spectrometer and Spectra Manager version 2.13.00 software. Samples were measured using continuous scan mode from 190 to 300 nm at 22°C, with a data pitch of 0.5 m, a scan speed of 50 nm/min, a CD scale of 200 mdeg/1.0 dOD, and three accumulation replicates. A buffer baseline was subtracted from all samples. Raw data were exported and plotted in KaleidaGraph.

### StNrdD activation and EPR spectroscopy quantification of glycyl radical

Inside an anaerobic chamber (MBRAUN), activation reactions were prepared in a clear Eppendorf tube, where 0.2 mM AdoMet, 50 μM 5-deazariboflavin (Santa Cruz Biotechnology), 1% glycerol, nucleotides (if present), 4 μM StNrdG monomer (added second to last), and 10 μM preincubated StNrdD dimer (added last) were diluted and mixed in 30 mM KCl and 30 mM bicine (pH 7.6). After the addition of StNrdD dimer, activation reactions were illuminated for 1 hour using a 2500-lumen light-emitting diode bulb and the MBRAUN chamber light. After, the entire reaction mixture was transferred into an EPR tube and frozen in liquid nitrogen before being taken out of the anaerobic chamber. Potassium nitrosodisulfonate (Fremy’s salt) was used as a radical standard for EPR quantification of glycyl radical. EPR spectra were collected in a Bruker EMXplus spectrometer equipped with a Bruker/ColdEdge 4 K waveguide cryogen-free cryostat. Spectra were recorded at 80 K, with a modulation amplitude of 3 G, a center field of 3350 G, a sweep width of 200 G, and a sweep time of 21 s at a microwave power of 52 dB. Each spectrum analyzed is an average of 10 scans. Xenon 1.1b.155 software was used to collect and process spectra. The double integrals of each spectrum were calculated using Xenon software and compared to the Fremy’s standard to determine the concentration of glycyl radical. Samples were normalized to the level of radical in the Apo-WT sample run on the same day. Spectra were graphed using Microsoft Excel version 16.90.2.

### Product turnover assays and LC-MS/MS analysis

Product turnover was measured using the anaerobic activity assays detailed in Levitz *et al.* (*31*), briefly summarized below with any changes described. In an anaerobic chamber (MBRAUN), following glycyl radical activation and determination of glycyl radical content, performed as described above, the concentration of StNrdD was adjusted to account for the day-of glycyl radical concentration. Then, protein master mixes, as previously described (*31*), were made using the amount of StNrdD to result in a final glycyl radical concentration of 0.15 μM in each assay. These steps were repeated for both WT-StNrdD and each variant. Nucleotide master mixes containing 1 mM substrate GTP, 1 mM specificity effector TTP, 3 mM allosteric activator ATP, or 3 mM allosteric inhibitor dATP were prepared and mixed separately. The addition of nucleotide master mix to protein master mix marked the initiation of the assay, which was incubated for a total of 120 s at 37°C before inactivation at 95°C for an additional 120 s. A total of 1 μl of 1.6 mM heavy dGTP (^13^C/^15^N) was added as an internal standard to each 40-μl sample and to the dGTP standard, which all were treated with calf intestinal alkaline phosphatase, filtered, and transferred to LC-MS plates. A standard curve was generated from a series of calibrations standards as described (*31*). Both samples and standards were run using an Agilent Ultivo triple quadrupole mass spectrometer coupled to an Agilent 1260 Infinity II LC system, using the methods as described (*31*). Data processing and integration to quantify total dGTP product made in each sample were completed using the Agilent MassHunter Qualitative Analysis program. Data were graphed using GraphPad Prism 9.5.1.

### Crystallization of WT-StNrdD with dATP

WT-StNrdD was purified as described above and concentrated to 33.5 mg/ml, in 20 mM Hepes (pH 7.6) and 100 mM sodium chloride. The His_6_ purification tag was cleaved with thrombin before crystallization. Initial conditions were identified in a ProComplex 96-well sparse matrix screening tray (Molecular Dimensions, currently available as ProPlex) dispensed by an Art Robbins Instruments Phoenix liquid-handling robot. Optimization of conditions identified in screening trays was attempted, but the best diffracting crystals came directly from the sitting drop 96-well screening tray. Crystals were grown at room temperature in 1.5 M ammonium sulfate and 0.1 M Hepes (pH 7.0), which was condition F12 in ProComplex tray. Crystals were cryoprotected, and nucleotides were introduced by looping through a solution of 15% glycerol, 1.5 M ammonium sulfate, 100 mM ammonium formate, 30 mM magnesium sulfate, 1 mM Tris(2-carboxyethyl)phosphine hydrochlorine (TCEP), 50 mM Hepes, 10 mM CTP, and 10 mM dATP and plunged directly in liquid N_2_.

### X-ray data collection

Diffraction data for WT-StNrdD-dATP were collected at the Advanced Photon Source on beamline 24-ID-C at 100 K. Diffraction data were indexed, integrated, and scaled using XDS. Data collection statistics are summarized in table S1.

### Crystal structure determination and refinement

The WT-StNrdD-dATP structure, with the space group of *P*2_1_2_1_2_1_ (cell dimensions: *a* = 90.580 Å, *b* = 97.885 Å, and *c* = 189.202 Å), was solved to a 2.60-Å resolution by molecular replacement in PHENIX (*45*), in the SBGrid software package (*46*), using the previously solved structure of T4 bacteriophage class III RNR (*17*) [Protein Data Bank (PDB): 1H79]. The asymmetric unit contained two molecules (one dimer). Multiple rounds of refinement were performed with phenix.refine (*45*). Refinement consisted of *XYZ* coordinate (reciprocal space) and individual B factor refinement. Noncrystallographic symmetry was not applied. Manual rebuilding and geometry correction were performed in Coot (*47*). Composite omit maps were used to validate the structure. Coordination distances for Mg^2+^ ions involved in activity site dATP binding were explicitly defined at 2.1 Å and with loose restraints. Coordination distances and angles for Mg^2+^ ions involved in specificity dATP binding were explicitly defined at 2.11 Å and 90°, respectively, and with loose restraints. Water and sulfate molecules were manually added in Coot. Chain A contains protein residues 8 to 133,138 to 669, and 676 to 696 (total of 676 residues of 734). Chain A also contains two Mg^2+^ ions, three dATP molecules, and one Zn^2+^ ion. Chain B contains protein residues 14 to 111 and 118 to 734 (total of 713 residues of 734). Chain B also contains two Mg^2+^ ions, three dATP molecules, and one Zn^2+^ ion. The structure contains 15 sulfate molecules and 156 water molecules. Refinement statistics for the WT dATP-StNrdD crystal structure are found in table S1. All structural figures were made in ChimeraX-1.6.1 (*48*).

### Cryo-EM sample preparation and data collection

#### StNrdD grid preparation and data acquisition

StNrdD was diluted to either 0.3 or 0.25 mg/ml and mixed with 1 mM GTP, 1 mM TTP, and either 3 mM ATP or 3 mM dATP in 30 mM tris (pH 7.5), 30 mM KCl, and 10 mM magnesium sulfate. Mixtures were incubated at 37°C for 2 min and then stored on ice before application to the grid. Quantifoil Cu 1.2/1.3 300-mesh grids were glow discharged for 60 s at −15- to −20-mA current. Grids were frozen on a Vitrobot Mark IV (Thermo Fisher Scientific) with the chamber humidity set to 95% and temperature set to 8°C. Three microliters of sample was applied to the grid and blotted for 5 s with a blot force of 5 before plunging into liquid ethane.

All cryo-EM datasets were collected at MIT.nano on a Titan Krios G3i (Thermo Fisher Scientific) operating at 300 kV with a Gatan K3 direct detector and BioQuantum image filter operating in super-resolution mode, at a nominal magnification of ×105,000 (resulting in a pixel size of 0.8324). Automated data collection was carried out with EPU (Thermo Fisher Scientific) with a nominal defocus range from −0.75 to −2.5 μm. The ATP-StNrdD dataset consisted of 12,463 exposures collected with a total dose of 51.35 e^−^ Å^−2^ over 30 fractions. The dATP-StNrdD dataset consisted of 21,675 exposures with a total dose of 51.43 e^−^ Å^−2^ over 30 fractions. All data collection parameters are summarized in table S2. All grids were screened for quality at 92× on a Talos Arctica G2 cryo–transmission electron microscopy (Thermo Fisher Scientific), operating at 200 kV with a Falcon 3EC direct electron detector before data collection.

### Cryo-EM data processing

Cryo-EM data processing was carried out using a combination of RELION-4.0.0 (*49*), CryoSPARC v3.3.2 (*50*, *51*), Warp (*52*), and LocSpiral (*53*) within the COSMIC2 science gateway (*54*). Structure refinements were carried out using PHENIX (*45*) and Coot (*47*). All structural figures were made in ChimeraX-1.6.1 (*48*). All programs, except for CryoSPARC and LocSpiral, were accessed via SBGrid (*46*). The processing strategy for each dataset is described below and summarized in figs. S5 and S6. Fourier shell correlation (FSC) curves are shown in fig. S7.

### Processing strategy

#### ATP-StNrdD

These data were processed, in parallel, with two different strategies to either improve core resolution or to obtain and improve cone domain density. Each strategy is described separately below, with a workflow shown in fig. S5.

Using RELION’s (*49*) implementation of MotionCor2 and CTFFind4.1, motion correction and CTF estimation were performed. Around 1000 particles were manually picked for each dataset from a subset of aligned movies and used to train a Topaz-based particle picker, using the Topaz wrapper in RELION. The trained Topaz network was used to pick particles across all micrographs. Particles were reextracted (box size, 126 pixels), and two rounds (25 iterations each) of reference-free 2D classification (mask diameter, 160 Å) were performed, each generating 200 classes.

Data were simultaneously processed in CryoSPARC (*50*) for use in a 3D variability analysis (3DVA) job. Micrographs were imported into CryoSPARC, and motion correction and CTF estimation were performed. Particles were picked using a trained blob picker, before use in 2D classification (50 classes). Two rounds of 2D classification (200 classes) were used to filter-picked particles before the selected particles (240,230 particles) were used in a multiclass reference-free ab initio reconstruction to generate six models. The particles from the best classes (166,344 particles) were used in a nonuniform refinement before inputting them into an intermediate 3DVA job with a 15-Å resolution filter that output particle subsets. Particles were combined from the “cone out” volumes of the 3DVA output (particles from frames 16 to 19 from component_000; 29,337 particles) and used in a nonuniform refinement. Particles were combined from the “core only” volumes of the 3DVA output (particles from frames 0 to 3 from component_000; 32,070 particles) and used in a nonuniform refinement.

These refined volumes were imported into RELION (*49*) for use as a reference for two 3D classifications, performed in parallel, to sort out particles containing clear cone domains or clear core density. Particles with the best core density were selected (146,371 particles) and used in masked 3D autorefinements, followed by postprocessing and CTF refinements. After CTF refinement, the particle polishing was performed using the Bayesian method of particle motion estimation in RELION. The polished particles were then used in another masked 3D autorefinement. Particles with the best cone density were selected (143,929 particles) and used in masked 3D autorefinements. A mask was generated to cover the cone domains and used in signal subtraction. The subtracted particles were used in 3D autorefinements, followed by CTF refinements. The separate subtracted cone particle map was combined with the polished core map using Frankenmap, integrated within Warp (*52*). Local B factor sharpening was performed using LocSpiral (*53*) within the COSMIC2 servers (*54*). Local resolution estimates for the final map is shown in fig. S8.

#### dATP-StNrdD

Using RELION’s (*49*) implementation of MotionCor2 and CTFFind4.1, motion correction and CTF estimation were performed. Around 1000 particles were manually picked for each dataset from a subset of aligned movies and used to train a Topaz-based particle picker, using the Topaz wrapper in RELION. The trained Topaz network was used to pick particles across all micrographs. Particles were reextracted (box size, 126 pixels), and two rounds (25 iterations each) of reference-free 2D classification (mask diameter, 160 Å) were performed, each generating 200 classes. Selected particles (3,659,249 particles) were processed, in parallel, with two different strategies to either improve cone domain density or improve core resolution. Each strategy is described separately below with a workflow shown in fig. S6.

With a focus on improving core resolution, selected particles (3,659,249 particles) were used to make an initial model in RELION. The RELION initial model was used as a reference in 3D classification. Classes with volumes containing clear core domain density were selected (942,509 particles). Following selection, the particles were reextracted (box size, 256 pixels) and used in a 3D autorefinement with the RELION initial model as reference and mask. A second round of 3D autorefinement was then done on the same particle stack, using the previous refinement as a reference and mask. Postprocessing was done, followed by CTF refinements. After CTF refinement, the particle polishing was performed using the Bayesian method of particle motion estimation in RELION. The polished particles (942,509 particles) were then used in another masked 3D autorefinement.

The selected particles were also imported into CryoSPARC (*50*) and used to make a reference free ab initio model. The CryoSPARC ab initio model was used as a reference in a RELION consensus refinement of all 2D selected particles. This refinement was then used as a reference in a 3D classification from which the best particles were selected (748,857 particles) and used in a 3D autorefinement. A mask was generated to cover the cone domains and used in signal subtraction. The subtracted particles were used in 3D autorefinements, followed by masked 3D classification. Particles with the best cone density were selected (478,351 particles) and used in masked 3D autorefinement, followed by CTF refinement and another round of masked 3D autorefinement.

The maps from each parallel processing strategy (separate subtracted cone map and polished core map) were combined using Frankenmap, integrated within Warp (*52*). Local B factor sharpening was performed using LocSpiral (*53*) within the COSMIC2 servers (*54*). Local resolution estimates for the final map are shown in fig. S8.

### Cryo-EM model refinement

phenix.dock_in_map (*45*) was used to dock coordinates from the crystal structure of StNrdD bound to dATP into each of the cryo-EM reconstructions. The model was refined through iterative rounds of phenix.real_space_refine and manual adjustment by hand and using the Real Space Refine function in Coot (*47*). Global minimization, atomic displacement parameters, local grid search, rigid body refinement, morphing (once per refinement), and simulated annealing (every macrocycle) were applied during initial rounds of PHENIX Real Space Refinement, and global minimization, atomic displacement parameters, local grid search, and morphing (once per refinement) were applied during final rounds of PHENIX Real Space Refinement.

In the aerobic ATP-StNrdD structure, chain A contains protein residues 1 to 119 and 138 to 727 (total of 707 residues of 734). ATP-StNrdD chain A also contains two Mg^2+^ ions, two ATP molecules, one TTP molecule, one GTP molecule, and three water molecules. ATP-StNrdD chain B contains protein residues 8 to 119 and 138 to 733 (total of 706 residues of 734). ATP-StNrdD chain B also contains two Mg^2+^ ions, two ATP molecules, one TTP molecule, one Zn^2+^ ion, and three water molecules. In the aerobic dATP-StNrdD structure, chain A contains protein residues 8 to 118, 138 to 671, and 675 to 728 (total of 696 residues of 734). dATP-StNrdD chain A also contains two Mg^2+^ ions, one dATP molecule, one TTP molecule, one Zn^2+^ ion, and three water molecules. dATP-StNrdD chain B contains protein residues 11 to 20, 24 to 117, and 138 to 733 (total of 697 residues of 734). dATP-StNrdD chain B contains two Mg^2+^ ions, two dATP molecule, one TTP molecule, one Zn^2+^ ion, and three water molecules. Refinement and validation statistics for both structures are found in table S2. All structural figures were made in ChimeraX-1.6.1 (*48*).

### HDX sample preparation

WT-StNrdD was diluted to 20 μM in 30 mM tris (pH 7.5), 30 mM potassium chloride, and 10 mM magnesium sulfate with the addition of either 1 mM GTP, 1 mM TTP or 1 mM GTP, 1 mM TTP, and either 3 mM ATP or 3 mM dATP. Samples were incubated at 37°C for 2 min and then kept on ice. The corresponding ^2^H_2_O buffers were prepared for each nucleotide condition and kept at room temperature. ^2^HCl and NaO^2^H were used to adjust the p^2^H to 7.5. ^2^H_2_O, ^2^HCl, and NaO^2^H were purchased from Cambridge Isotope Laboratories. Quench buffer consisting of 8 M urea, 100 mM glycine, and 200 mM TCEP, (pH 2.5) and dilution buffer consisting of 100 mM glycine and 200 mM TCEP (pH 2.5) were both prepared and kept on ice. Two microliters of each protein sample was equilibrated at room temperature for 5 min before addition of 18 μl of H_2_O buffer or ^2^H2O buffer for undeuterated control samples and HDX reactions samples, respectively. For the HDX reactions, samples were incubated for either 20 s, 1 min, 10 min, 1 hour, or 2 hours following the addition of ^2^H_2_O buffer. Subsequently, 80 μl of quench buffer was immediately added, and the sample was incubated on ice for 1 min before the addition of 150 μl of dilution buffer. Samples were then frozen in liquid nitrogen and stored at −80°C. Deuterated samples were prepared for three protein states (no activity effector, ATP-bound, and dATP-bound) across five time points (20 s, 1 min, 10 min, 1 hour, and 2 hours). For each nucleotide condition, all time points were completed in triplicate and prepared on the same day.

### HDX data acquisition and analysis

Immediately after thawing, 25 μl of the solution was injected into a Waters M-Class ultraperformance LC system equipped with HDX technology and online digestion with an acid protease column. The protease column was prepared in-house by packing a C-130B guard column with pepsin immobilized onto agarose resin beads (Thermo Fisher Scientific). Peptic fragments were trapped on an ACQUITY VanGuard precolumn (BEH C18, 1.7 μm, 2.1 mm by 5 mm) and separated with an ACQUITY UPLC column (BEH C18, 1.7 μm, 1.0 mm by 100 mm) using a 7-min gradient of solvent B (0.1% formic acid in acetonitrile) increasing linearly from 5 to 35% at a flow rate of 40 μl/min. The peptides were then eluted into a Waters Synapt G2Si mass spectrometer. MS data were acquired using HDMS^E^ with ion mobility separation and continuous lock (Leu-Enk) for mass accuracy correction. A capillary voltage of 3.0 kV and a 20- to 30-V ramp trap collisional energy for high-energy acquisition of product ions were used. Peptides were identified from the undeuterated control samples via Waters’ ProteinLynx Global Server 3.0.3. Mass error tolerance was set to 10 parts per million (ppm) for precursor peptides and 20 ppm for product ions. Further filtering of 0.3 fragments per residue was applied in Waters’ DynamX 3.0 to determine final sequence coverage map. HDX reactions were acquired as above with one exception, 50 μl of each sample was injected instead of 25 μl to improve the signal-to-noise ratio. DynamX 3.0 was used to curate isotopic envelops, determine envelop centroids, and assess the absolute deuterium uptake for each peptide as a function of labeling time. Sequence coverage was 98.8% with 6.28 redundancy.

## References

[1] N. C. Brown, P. Reichard, Role of effector binding in allosteric control of ribonucleoside diphosphate reductase. J. Mol. Biol. 46, 39–55 (1969). 4902212 10.1016/0022-2836(69)90056-4

[2] P. Nordlund, P. Reichard, Ribonucleotide reductases. Annu. Rev. Biochem. 75, 681–706 (2006). 16756507 10.1146/annurev.biochem.75.103004.142443

[3] C. K. Mathews, DNA precursor metabolism and genomic stability. FASEB J. 20, 1300–1314 (2006). 16816105 10.1096/fj.06-5730rev

[4] L. J. Wheeler, I. Rajagopal, C. K. Mathews, Stimulation of mutagenesis by proportional deoxyribonucleoside triphosphate accumulation in *Escherichia coli*. DNA Repair 4, 1450–1456 (2005). 16207537 10.1016/j.dnarep.2005.09.003

[5] B. A. Kunz, S. E. Kohalmi, T. A. Kunkel, C. K. Mathews, E. M. McIntosh, J. A. Reidy, Deoxyribonucleoside triphosphate levels: A critical factor in the maintenance of genetic stability. Mutat. Res. 318, 1–64 (1994). 7519315 10.1016/0165-1110(94)90006-x

[6] B. L. Greene, G. Kang, C. Cui, M. Bennati, D. G. Nocera, C. L. Drennan, J. Stubbe, Ribonucleotide reductases (RNRs): Structure, chemistry, and metabolism suggest new therapeutic targets. Annu. Rev. Biochem. 89, 45–75 (2020). 32569524 10.1146/annurev-biochem-013118-111843 PMC7316142

[7] T. B. Ruskoski, A. K. Boal, The periodic table of ribonucleotide reductases. J. Biol. Chem. 297, 101137 (2021). 34461093 10.1016/j.jbc.2021.101137 PMC8463856

[8] L. R. F. Backman, M. A. Funk, C. D. Dawson, C. L. Drennan, New tricks for the glycyl radical enzyme family. Crit. Rev. Biochem. Mol. Biol. 52, 674–695 (2017). 28901199 10.1080/10409238.2017.1373741 PMC5911432

[9] R. Eliasson, M. Fontecave, H. Jörnvall, M. Krook, E. Pontis, P. Reichard, The anaerobic ribonucleoside triphosphate reductase from Escherichia coli requires S-adenosylmethionine as a cofactor. Proc. Natl. Acad. Sci. U.S.A. 87, 3314–3318 (1990). 2185465 10.1073/pnas.87.9.3314 PMC53890

[10] E. Torrents, Ribonucleotide reductases: Essential enzymes for bacterial life. Front. Cell Infect. Microbiol. 4, 52 (2014). 24809024 10.3389/fcimb.2014.00052 PMC4009431

[11] L. M. Bush, M. T. Vazquez-Pertejo, “Overview of anaerobic bacteria” (Merck Manuals Professional Edition, 2023); www.merckmanuals.com/professional/infectious-diseases/anaerobic-bacteria/overview-of-anaerobic-bacteria.

[12] X. Sun, R. Eliasson, E. Pontis, J. Andersson, G. Buist, B. M. Sjöberg, P. Reichard, Generation of the glycyl radical of the anaerobic Escherichia coli ribonucleotide reductase requires a specific activating enzyme. J. Biol. Chem. 270, 2443–2446 (1995). 7852304 10.1074/jbc.270.6.2443

[13] S. Ollagnier, E. Mulliez, P. P. Schmidt, R. Eliasson, J. Gaillard, C. Deronzier, T. Bergman, A. Gräslund, P. Reichard, M. Fontecave, Activation of the anaerobic ribonucleotide reductase from *Escherichia coli*. The essential role of the iron-sulfur center for S-adenosylmethionine reduction. J. Biol. Chem. 272, 24216–24223 (1997). 9305874 10.1074/jbc.272.39.24216

[14] J. L. Vey, J. Yang, M. Li, W. E. Broderick, J. B. Broderick, C. L. Drennan, Structural basis for glycyl radical formation by pyruvate formate-lyase activating enzyme. Proc. Natl. Acad. Sci. U.S.A. 105, 16137–16141 (2008). 18852451 10.1073/pnas.0806640105 PMC2571006

[15] P. Young, J. Andersson, M. Sahlin, B. M. Sjöberg, Bacteriophage T4 anaerobic ribonucleotide reductase contains a stable glycyl radical at position 580. J. Biol. Chem. 271, 20770–20775 (1996). 8702830 10.1074/jbc.271.34.20770

[16] S. J. Elledge, Z. Zhou, J. B. Allen, T. A. Navas, DNA damage and cell cycle regulation of ribonucleotide reductase. Bioessays 15, 333–339 (1993). 8343143 10.1002/bies.950150507

[17] K. M. Larsson, J. Andersson, B. M. Sjöberg, Structural basis for allosteric substrate specificity regulation in anaerobic ribonucleotide reductases. Structure 9, 739–750 (2001). 11587648 10.1016/s0969-2126(01)00627-x

[18] R. Eliasson, E. Pontis, X. Sun, P. Reichard, Allosteric control of the substrate specificity of the anaerobic ribonucleotide reductase from *Escherichia coli*. J. Biol. Chem. 269, 26052–26057 (1994). 7929317

[19] E. Torrents, G. Buist, A. Liu, R. Eliasson, J. Kok, I. Gibert, A. Gräslund, P. Reichard, The anaerobic (class III) ribonucleotide reductase from *Lactococcus lactis*. J. Biol. Chem. 275, 2463–2471 (2000). 10644700 10.1074/jbc.275.4.2463

[20] R. Rofougaran, M. Crona, M. Vodnala, B.-M. Sjöberg, A. Hofer, Oligomerization status directs overall activity regulation of the *Escherichia coli* class Ia ribonucleotide reductase. J. Biol. Chem. 283, 35310–35318 (2008). 18835811 10.1074/jbc.M806738200

[21] N. Ando, E. J. Brignole, C. M. Zimanyi, M. A. Funk, K. Yokoyama, F. J. Asturias, J. A. Stubbe, C. L. Drennan, Structural interconversions modulate activity of *Escherichia coli* ribonucleotide reductase. Proc. Natl. Acad. Sci. U.S.A. 108, 21046–21051 (2011). 22160671 10.1073/pnas.1112715108 PMC3248520

[22] M. A. Funk, C. M. Zimanyi, G. A. Andree, A. E. Hamilos, C. L. Drennan, How ATP and dATP act as molecular switches to regulate enzymatic activity in the prototypical bacterial class Ia ribonucleotide reductase. Biochemistry 63, 2517–2531 (2024). 39164005 10.1021/acs.biochem.4c00329 PMC11447812

[23] D. Lundin, G. Berggren, D. T. Logan, B.-M. Sjöberg, The origin and evolution of ribonucleotide reduction. Life 5, 604–636 (2015). 25734234 10.3390/life5010604 PMC4390871

[24] G. Kang, A. T. Taguchi, J. A. Stubbe, C. L. Drennan, Structure of a trapped radical transfer pathway within a ribonucleotide reductase holocomplex. Science 368, 424–427 (2020). 32217749 10.1126/science.aba6794 PMC7774503

[25] E. J. Brignole, K. L. Tsai, J. Chittuluru, H. Li, Y. Aye, P. A. Penczek, J. A. Stubbe, C. L. Drennan, F. Asturias, 3.3-Å resolution cryo-EM structure of human ribonucleotide reductase with substrate and allosteric regulators bound. eLife 7, e31502 (2018). 29460780 10.7554/eLife.31502 PMC5819950

[26] M. Eriksson, U. Uhlin, S. Ramaswamy, M. Ekberg, K. Regnström, B. M. Sjöberg, H. Eklund, Binding of allosteric effectors to ribonucleotide reductase protein R1: Reduction of active-site cysteines promotes substrate binding. Structure 5, 1077–1092 (1997). 9309223 10.1016/s0969-2126(97)00259-1

[27] C. M. Zimanyi, P. Y. T. Chen, G. Kang, M. A. Funk, C. L. Drennan, Molecular basis for allosteric specificity regulation in class Ia ribonucleotide reductase from Escherichia coli. eLife 5, e07141 (2016). 26754917 10.7554/eLife.07141 PMC4728125

[28] O. Bimaï, I. Banerjee, I. R. Grinberg, P. Huang, D. Lundin, B.-M. Sjöberg, D. T. Logan, Activity modulation in anaerobic ribonucleotide reductases: Nucleotide binding to the ATP-cone mediates long-range order-disorder transitions in the active site. eLife 12, RP89292 (2023).

[29] O. Uriot, S. Denis, M. Junjua, Y. Roussel, A. Dary-Mourot, S. Blanquet-Diot, *Streptococcus thermophilus*: From yogurt starter to a new promising probiotic candidate? J. Funct. Foods 37, 74–89 (2017).

[30] R. Iyer, S. K. Tomar, T. U. Maheswari, R. Singh, *Streptococcus thermophilus* strains: Multifunctional lactic acid bacteria. Int. Dairy J. 20, 133–141 (2010).

[31] T. S. Levitz, G. A. Andree, R. Jonnalagadda, C. D. Dawson, R. E. Bjork, C. L. Drennan, A rapid and sensitive assay for quantifying the activity of both aerobic and anaerobic ribonucleotide reductases acting upon any or all substrates. PLOS ONE 17, e0269572 (2022). 35675376 10.1371/journal.pone.0269572 PMC9176816

[32] I. Rozman Grinberg, M. Martínez-Carranza, O. Bimai, G. Nouaïria, S. Shahid, D. Lundin, D. T. Logan, B. M. Sjöberg, P. Stenmark, A nucleotide-sensing oligomerization mechanism that controls NrdR-dependent transcription of ribonucleotide reductases. Nat. Commun. 13, 2700 (2022). 35577776 10.1038/s41467-022-30328-1 PMC9110341

[33] I. R. Grinberg, D. Lundin, M. Hasan, M. Crona, V. R. Jonna, C. Loderer, M. Sahlin, N. Markova, I. Borovok, G. Berggren, A. Hofer, D. T. Logan, B.-M. Sjöberg, Novel ATP-cone-driven allosteric regulation of ribonucleotide reductase via the radical-generating subunit. eLife 7, e31529 (2018). 29388911 10.7554/eLife.31529 PMC5794259

[34] D. Rehling, E. R. Scaletti, I. Rozman Grinberg, D. Lundin, M. Sahlin, A. Hofer, B.-M. Sjöberg, P. Stenmark, Structural and biochemical investigation of class I ribonucleotide reductase from the hyperthermophile *Aquifex aeolicus*. Biochemistry 61, 92–106 (2022). 34941255 10.1021/acs.biochem.1c00503 PMC8772380

[35] V. R. Jonna, M. Crona, R. Rofougaran, D. Lundin, S. Johansson, K. Brännström, B.-M. Sjöberg, A. Hofer, Diversity in overall activity regulation of ribonucleotide reductase. J. Biol. Chem. 290, 17339–17348 (2015). 25971975 10.1074/jbc.M115.649624 PMC4498072

[36] R. Johansson, V. R. Jonna, R. Kumar, N. Nayeri, D. Lundin, B.-M. Sjöberg, A. Hofer, D. T. Logan, Structural mechanism of allosteric activity regulation in a ribonucleotide reductase with double ATP cones. Structure 24, 906–917 (2016). 27133024 10.1016/j.str.2016.03.025

[37] T. S. Levitz, E. J. Brignole, I. Fong, M. C. Darrow, C. L. Drennan, Effects of chameleon dispense-to-plunge speed on particle concentration, complex formation, and final resolution: A case study using the *Neisseria gonorrhoeae* ribonucleotide reductase inactive complex. J. Struct. Biol. 214, 107825 (2022). 34906669 10.1016/j.jsb.2021.107825 PMC8994553

[38] M. Martínez-Carranza, V. R. Jonna, D. Lundin, M. Sahlin, L. A. Carlson, N. Jemal, M. Högbom, B.-M. Sjöberg, P. Stenmark, A. Hofer, A ribonucleotide reductase from *Clostridium botulinum* reveals distinct evolutionary pathways to regulation via the overall activity site. J. Biol. Chem. 295, 15576–15587 (2020). 32883811 10.1074/jbc.RA120.014895 PMC7667963

[39] J. W. Fairman, S. R. Wijerathna, M. F. Ahmad, H. Xu, R. Nakano, S. Jha, J. Prendergast, R. M. Welin, S. Flodin, A. Roos, P. Nordlund, Z. Li, T. Walz, C. G. Dealwis, Structural basis for allosteric regulation of human ribonucleotide reductase by nucleotide-induced oligomerization. Nat. Struct. Mol. Biol. 18, 316–322 (2011). 21336276 10.1038/nsmb.2007 PMC3101628

[40] H. Xu, C. Faber, T. Uchiki, J. Racca, C. Dealwis, Structures of eukaryotic ribonucleotide reductase I define gemcitabine diphosphate binding and subunit assembly. Proc. Natl. Acad. Sci. U.S.A. 103, 4028–4033 (2006). 16537480 10.1073/pnas.0600440103 PMC1389703

[41] J. Narasimhan, S. Letinski, S. P. Jung, A. Gerasyuto, J. Wang, M. Arnold, G. Chen, J. Hedrick, M. Dumble, K. Ravichandran, T. Levitz, C. Cui, C. L. Drennan, J. A. Stubbe, G. Karp, A. Branstrom, Ribonucleotide reductase, a novel drug target for gonorrhea. eLife 11, e67447 (2022). 35137690 10.7554/eLife.67447 PMC8865847

[42] W. van Schaik, The human gut resistome. Philos. Trans. R Soc. Lond. B Biol. Sci. 370, 20140087 (2015). 25918444 10.1098/rstb.2014.0087 PMC4424436

[43] T. P. Stinear, D. C. Olden, P. D. R. Johnson, J. K. Davies, M. L. Grayson, Enterococcal *vanB* resistance locus in anaerobic bacteria in human faeces. Lancet 357, 855–856 (2001). 11265957 10.1016/S0140-6736(00)04206-9

[44] D. T. Logan, J. Andersson, B.-M. Sjöberg, P. Nordlund, A glycyl radical site in the crystal structure of a class III ribonucleotide reductase. Science 283, 1499–1504 (1999). 10066165 10.1126/science.283.5407.1499

[45] P. D. Adams, R. W. Grosse-Kunstleve, L.-W. Hung, T. R. Ioerger, A. J. McCoy, N. W. Moriarty, R. J. Read, J. C. Sacchettini, N. K. Sauter, T. C. Terwilliger, PHENIX: Building new software for automated crystallographic structure determination. Acta Crystallogr. D Biol. Crystallogr. 58, 1948–1954 (2002). 12393927 10.1107/s0907444902016657

[46] A. Morin, B. Eisenbraun, J. Key, P. C. Sanschagrin, M. A. Timony, M. Ottaviano, P. Sliz, Collaboration gets the most out of software. eLife 2, e01456 (2013). 24040512 10.7554/eLife.01456 PMC3771563

[47] P. Emsley, B. Lohkamp, W. G. Scott, K. Cowtan, Features and development of Coot. Acta Crystallogr. D Biol. Crystallogr. 66, 486–501 (2010). 20383002 10.1107/S0907444910007493 PMC2852313

[48] E. F. Pettersen, T. D. Goddard, C. C. Huang, E. C. Meng, G. S. Couch, T. I. Croll, J. H. Morris, T. E. Ferrin, UCSF ChimeraX: Structure visualization for researchers, educators, and developers. Prot. Sci. 30, 70–82 (2021). 10.1002/pro.3943 PMC7737788 32881101

[49] J. Zivanov, J. Otón, Z. Ke, A. von Kügelgen, E. Pyle, K. Qu, D. Morado, D. Castaño-Díez, G. Zanetti, T. A. M. Bharat, J. A. G. Briggs, S. H. W. Scheres, A Bayesian approach to single-particle electron cryo-tomography in RELION-4.0. eLife 11, e83724 (2022). 36468689 10.7554/eLife.83724 PMC9815803

[50] A. Punjani, J. L. Rubinstein, D. J. Fleet, M. A. Brubaker, cryoSPARC: Algorithms for rapid unsupervised cryo-EM structure determination. Nat. Methods 14, 290–296 (2017). 28165473 10.1038/nmeth.4169

[51] A. Punjani, D. J. Fleet, 3D variability analysis: Resolving continuous flexibility and discrete heterogeneity from single particle cryo-EM. J. Struct. Biol. 213, 107702 (2021). 33582281 10.1016/j.jsb.2021.107702

[52] D. Tegunov, P. Cramer, Real-time cryo-electron microscopy data preprocessing with Warp. Nat. Methods 16, 1146–1152 (2019). 31591575 10.1038/s41592-019-0580-y PMC6858868

[53] S. Kaur, J. Gomez-Blanco, A. A. Z. Khalifa, S. Adinarayanan, R. Sanchez-Garcia, D. Wrapp, J. S. McLellan, K. H. Bui, J. Vargas, Local computational methods to improve the interpretability and analysis of cryo-EM maps. Nat. Commun. 12, 1240 (2021). 33623015 10.1038/s41467-021-21509-5 PMC7902670

[54] M. A. Cianfrocco, M. Wong-Barnum, C. Youn, R. Wagner, A. Leschziner, “COSMIC2: A science gateway for cryo-electron microscopy structure determination,” in *PEARC '17: Practice and Experience in Advanced Research Computing 2017: Sustainability, Success and Impact* (Association for Computing Machinery, 2017), pp. 1–5.

