# Supplementary material for: How ATP and dATP reposition class III ribonucleotide reductase cone domains to regulate enzyme activity
Source: Sci Adv. 2025 Nov 28;11(48):eady9156. doi: 10.1126/sciadv.ady9156 (PMC12662201; doi:10.1126/sciadv.ady9156)
Supplement: Supplementary file 1 — Figs. S1 to S19 Tables S1 to S3 [file sciadv.ady9156_sm.pdf]

Science Advances
AAAS

Supplementary Materials for

How ATP and dATP reposition class III ribonucleotide reductase cone domains to regulate enzyme activity

Gisele A. Andree *et al.*

Corresponding author: Catherine L. Drennan, cdrennan@mit.edu

Sci. Adv. **11**, eady9156 (2025)
DOI: 10.1126/sciadv.ady9156

This PDF file includes:

Figs. S1 to S19
Tables S1 to S3

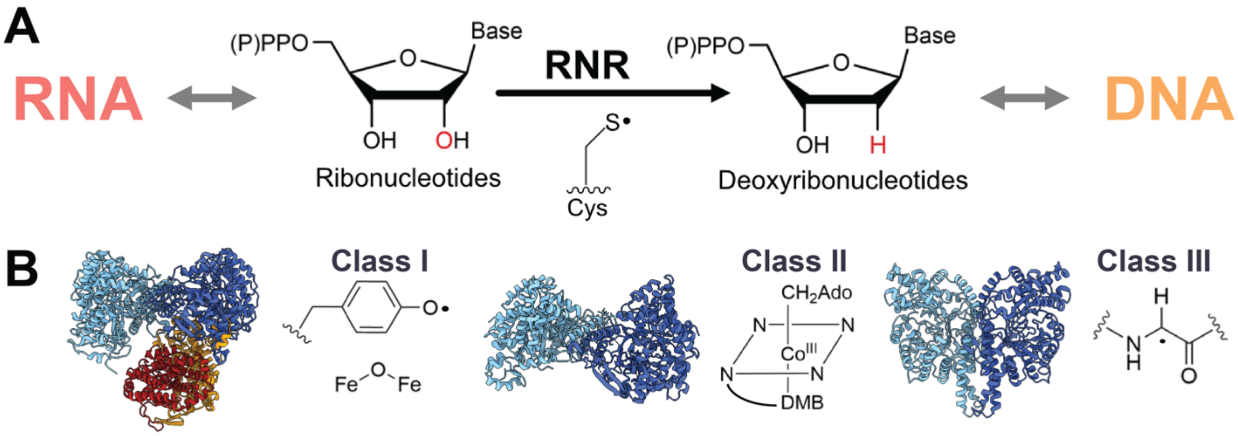

Fig. S1. Overview of ribonucleotide reductase reaction and classes. A) Ribonucleotide reductase (RNR) catalyzes the reduction of ribonucleotides to deoxyribonucleotides via a transient catalytic thiyl radical species. B) Examples of the main three classes of RNR and the cofactors used to generate the transient catalytic thiyl radical species. The class Ia RNR from *Escherichia coli* (left) is shown in the active form α2β2 conformation. The catalytic α subunits are shown in blue and the radical-generating β subunits shown in orange/red (PDB: 6W4X). To generate the catalytic thiyl radical species, class Ia RNR uses an oxygen dependent di-iron tyrosyl radical cofactor, which is located on the radical-storage β subunit. The class II RNR from *Thermotoga maritima* (middle) is shown in blue (PDB: 3O0N). An adenosylcobalamin cofactor is used to generate the thiyl radical species. The class III RNR from *Escherichia coli* bacteriophage T4 (right) is shown in blue (PDB: 1HK8). Class III RNRs utilize the glycyl radical cofactor that is installed on the catalytic subunit by the activase NrdG.

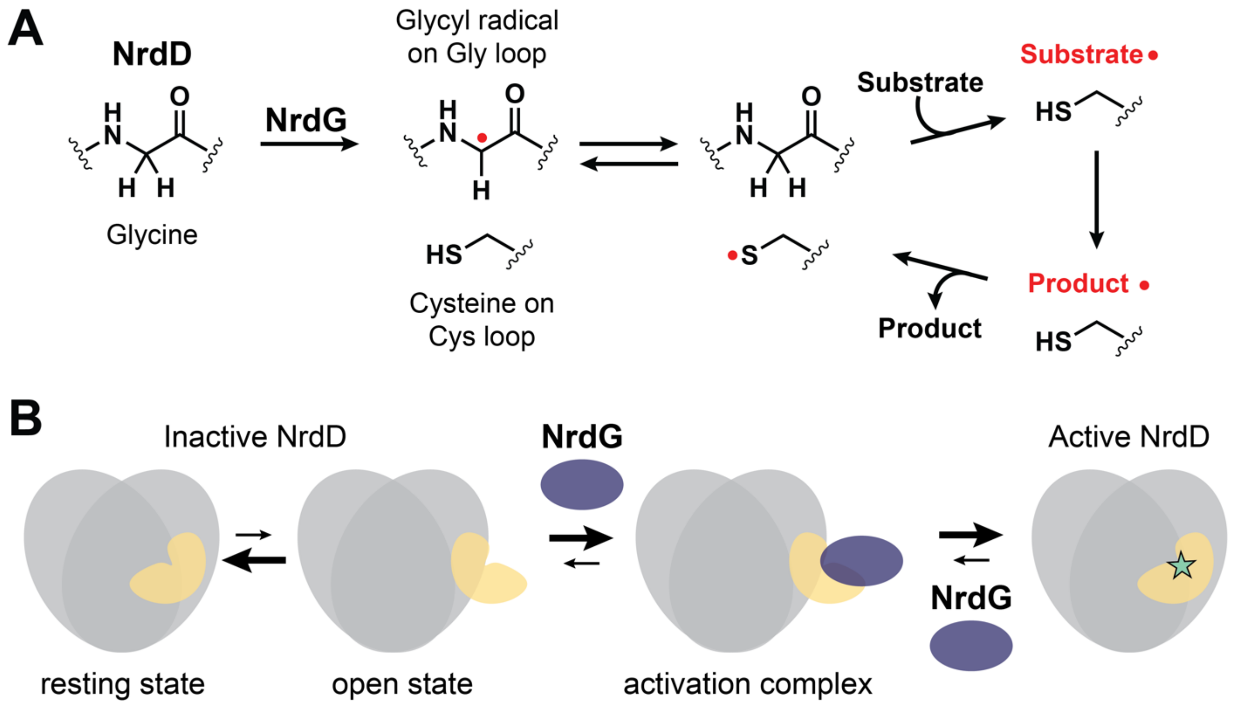

Fig. S2. Overview of glycyl radical installation and radical-based catalysis in class III RNR. A) Schematic of glycyl radical installation and turnover cycle. NrdG, a member of the radical SAM enzyme superfamily, requires an [4Fe-4S] cluster, *S*-adenosylmethionine (AdoMet), and an electron to install a glycyl radical species on the class III RNR enzyme NrdD. After the glycyl radical is installed on NrdD by the NrdG activase, the radical is transferred to the catalytic Cys, forming the catalytically essential thiyl radical species. The thiyl radical abstracts a hydrogen atom from substrate, forming a substrate-based radical species and then a product-based radical species. The thiyl radical species is re-formed, followed by the return of the radical to its storage position on the glycine of the Gly loop. B) Cartoon scheme of glycyl radical installation on the NrdD enzyme (grey) via interaction with the radical SAM activase, NrdG (purple). NrdG can only bind the open state of NrdD where the GRD (yellow) is flipped out. After forming the transient activation complex, NrdG dissociates and leaves the glycyl radical (green star) on the now active NrdD.

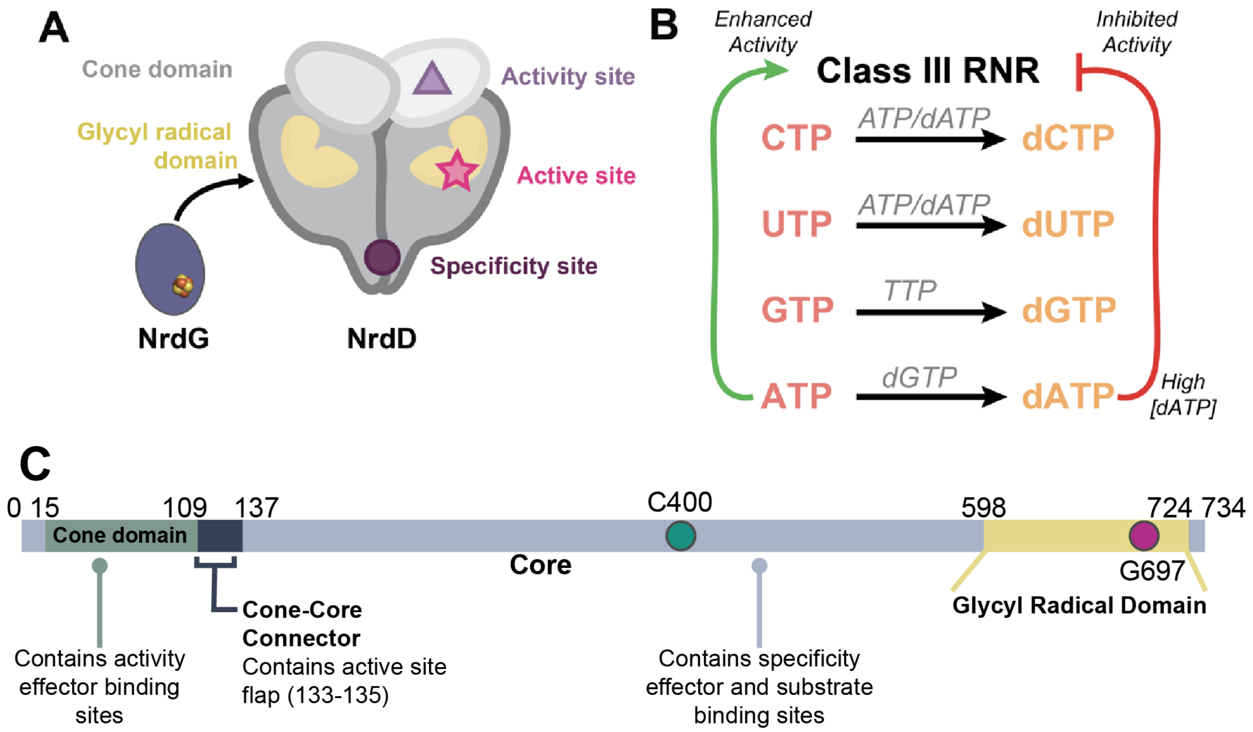

Fig. S3. Overview of class III RNR allosteric binding sites and regulation. A) Cartoon Fig. of NrdD (grey) showing allosteric sites, active sites and glycyl radical domains (GRDs, yellow). The radical SAM activase NrdG (purple oval) that installs the glycyl radical is also shown. There are three nucleotide binding sites on NrdD. The activity site (purple triangle), where ATP or dATP can bind to either enhance or inhibit enzymatic activity, respectively, is located in the N-terminal region of NrdD called the cone domain (light grey oval). The substrate binds in the active site (pink star), within the protein core. The class III RNR specificity site (purple circle), where specificity effectors bind to control which substrate gets reduced, is located along the dimer interface. B) Allosteric regulation rules for class III RNR. Specific substrate (coral text)-specificity effector (grey text) pairs are highlighted with the resulting product (orange text). ATP acts as an overall positive activity effector and dATP at high concentrations acts as an overall negative activity effector. C) Domain map of the class III RNR from *Streptococcus thermophilus* with nucleotide binding sites and thiyl radical Cys400 and glycyl radical Gly697 indicated. Panel duplicated from main text.

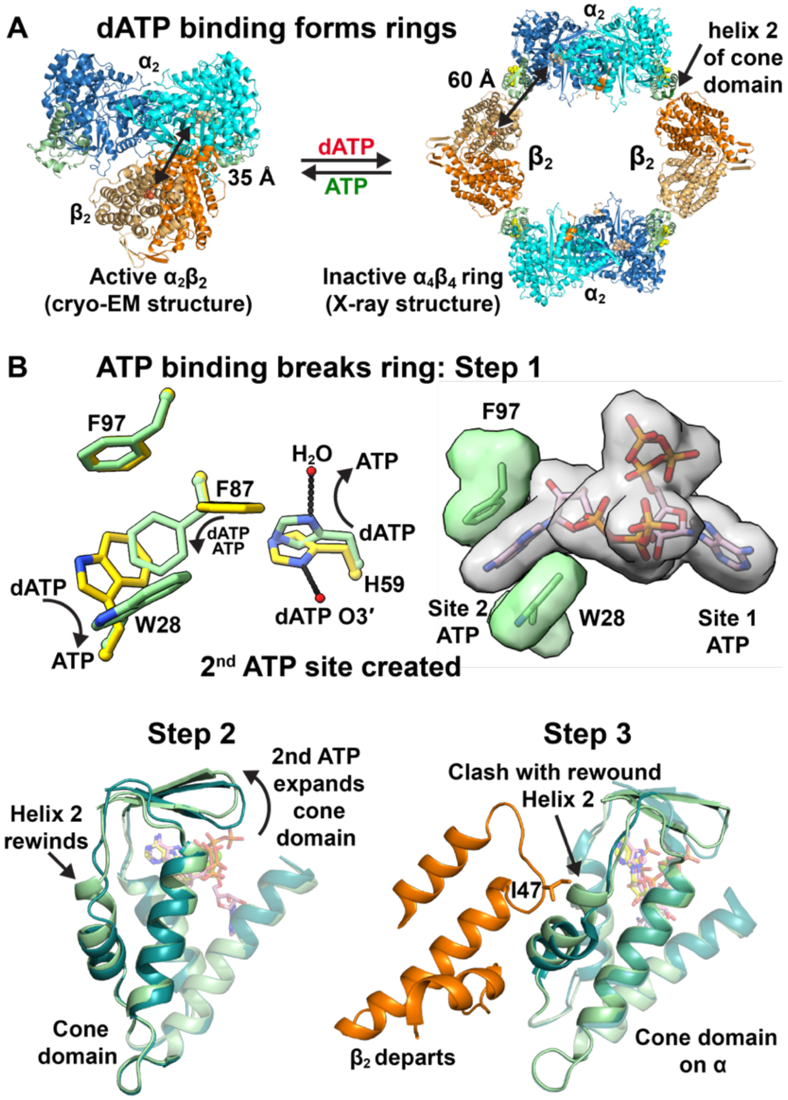

Fig. S4. Allosteric activity regulation in *Escherichia coli* class Ia RNR. A) Allosteric regulation of activity involves an oligomeric state change in *Escherichia coli* class Ia RNR. dATP binding to cone domain (green) leads to formation of an α4β4 ring structure (PDB: 5CNS) in which β2 (orange/brown) is too far (60 Å) from α2 (blue/cyan) for radical transfer. ATP binding to the cone domain shifts the equilibrium back to the active α2β2 state (PDB: 6W4X) that is capable of the 35-Å radical transfer. B) The molecular mechanism by which ATP breaks the ring and shifts the equilibrium toward the active state has been established for *E. coli* class Ia RNR. In the first step of this mechanism, a second nucleotide binding site is created in the cone domain. When ATP displaces dATP in site 1 of the cone domain, a hydrogen bond is lost to His59. The side chain of His59 moves, which causes the movement of Phe87, which in turn causes the Trp28 side chain to move, which creates the second ATP site. Residue positions from ATP structure in green and from dATP structure in yellow. Step 2: the binding of the second ATP to the cone domain expands the cone domain, which relieves a strain on helix 2. Helix 2 rewinds by one turn. Step 3: the rewinding of helix 2 creates a close contact with β2, and β2 departs.

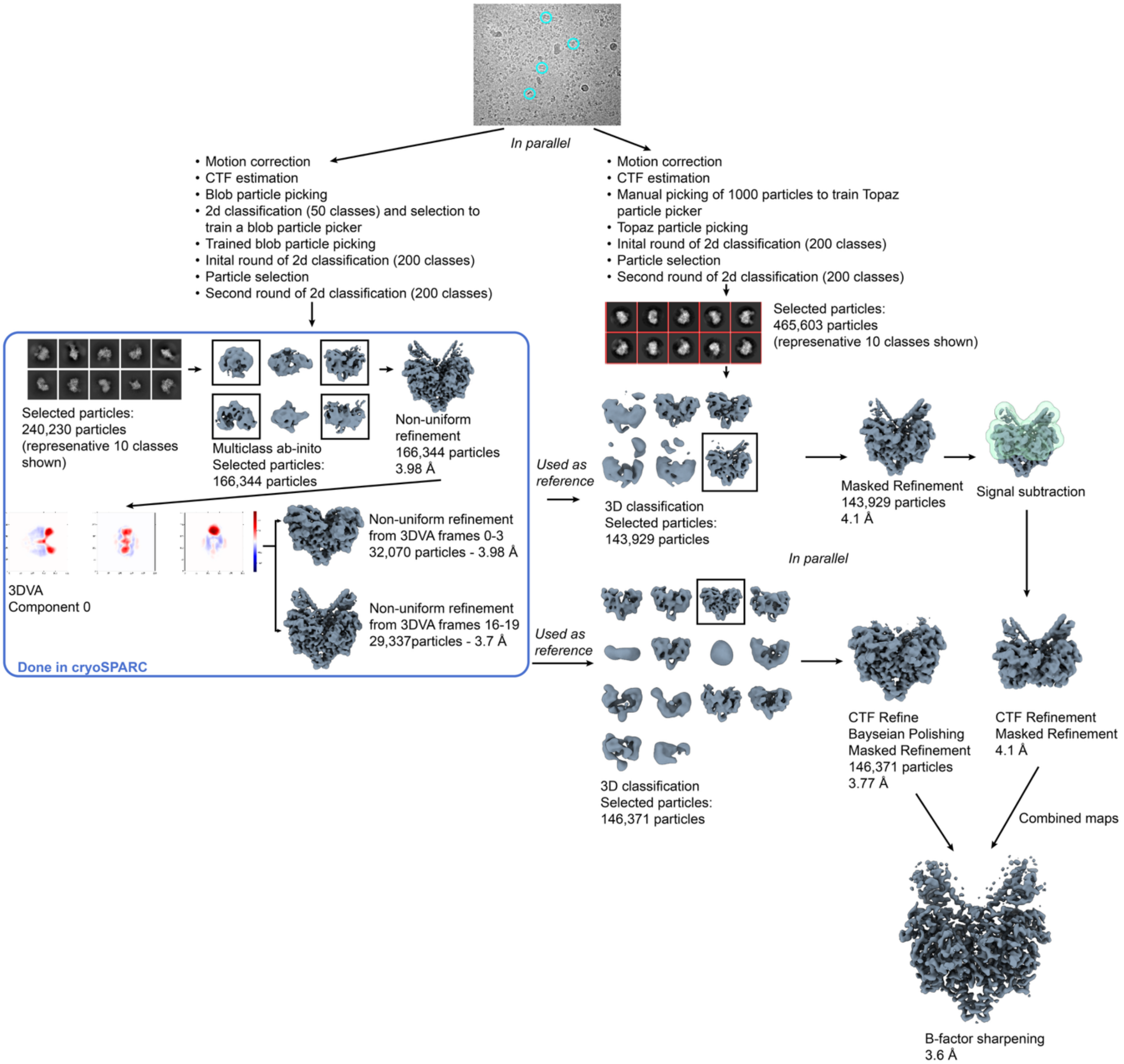

Fig. S5. Processing workflow for ATP-StNrdD dataset. Data were initially processed simultaneously in CryoSPARC and RELION. Representative particles are circled in cyan. Particles were picked using Topaz in RELION (465,603 particles after 2D classification) and using a trained blob picker in CryoSPARC. In CryoSPARC, selected particles (240,230 particles) were filtered using a multiclass ab-initio before refining and use in 3D variability analysis (3DVA). The particles subsets from the initial frames (0-3, 32,070 particles) and final frames (16-19, 29,337 particles) of 3DVA component 0 were selected and refined. These volumes were used as references only in parallel RELION in 3D classifications to pull out volumes with the best core volume (143,929 particles), or cone volume (146,371 particles). To improve cone density, signal was subtracted outside of the masked region containing the cone domains (green mask) and these particles were refined further. Each separate map was combined using Frankenmap and B-factor sharpening was applied using LocSprial.

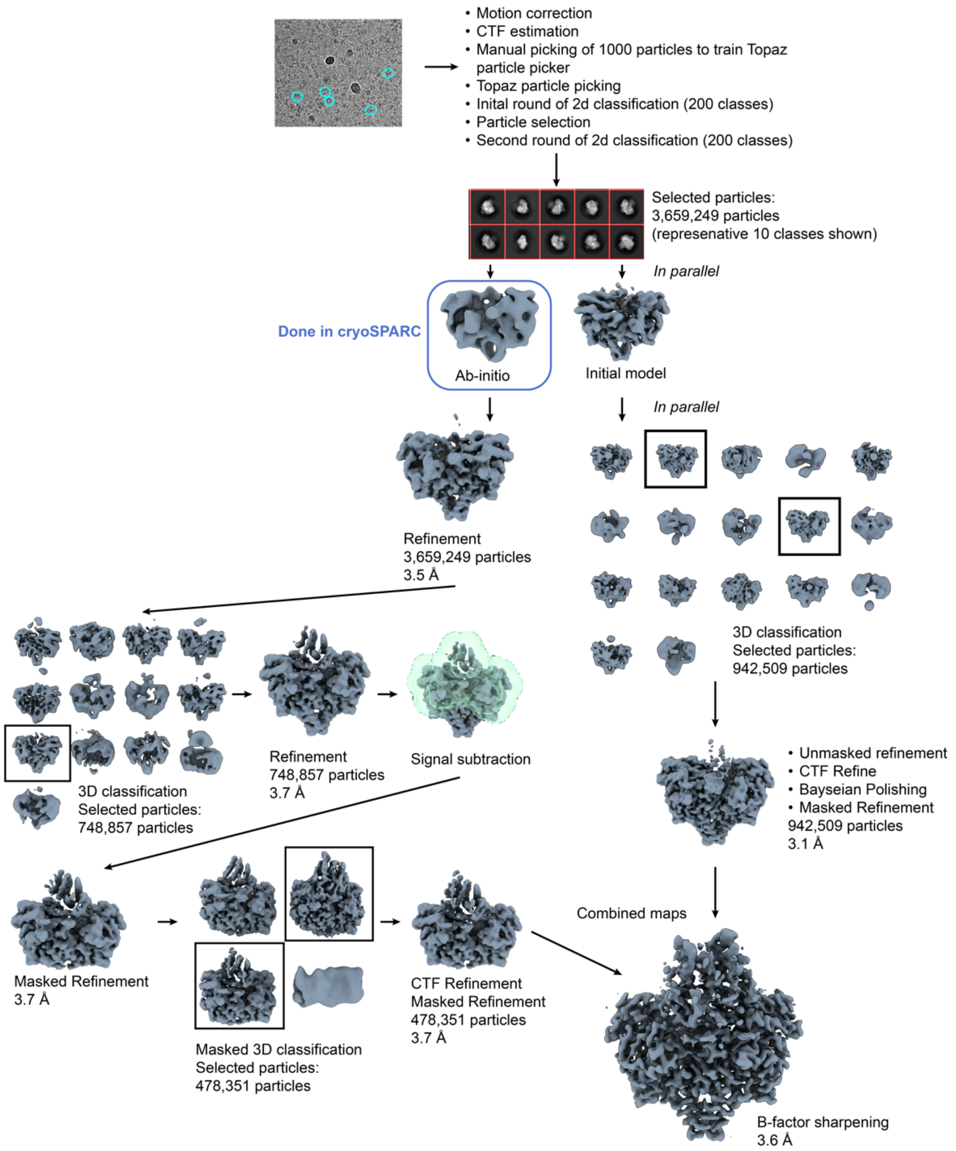

Fig. S6. Processing workflow for dATP-StNrdD dataset. Data were initially processed in RELION, representative particles are circled in cyan. Particles were picked using Topaz in RELION and filtered with 2D classification. These particles (3,659,249 particles) were transferred to CryoSPARC to create an ab-initio model. This model was used as a reference for a consensus refinement. This refinement was used as a reference in a 3D classification from which the particles in the black outline were moved forward (748,857 particles). To improve cone density, signal was subtracted outside of the masked region containing the cone domains (green mask) and these particles were refined further and used in masked 3D classifications, from which the particles in the black outlines were moved forward (478,351 particles). These particles were used in CTF refinement and masked refinement. In parallel, the original selection of particles (3,659,249 particles) was also used to generate an initial model in RELION before a 3D classification, from which the particles in the black outlines were moved forward (942,509 particles), and used in CTF refinement, Bayesian polishing, and masked refinements. Each separate map was combined using Frankenmap and B-factor sharpening was applied using LocSprial.

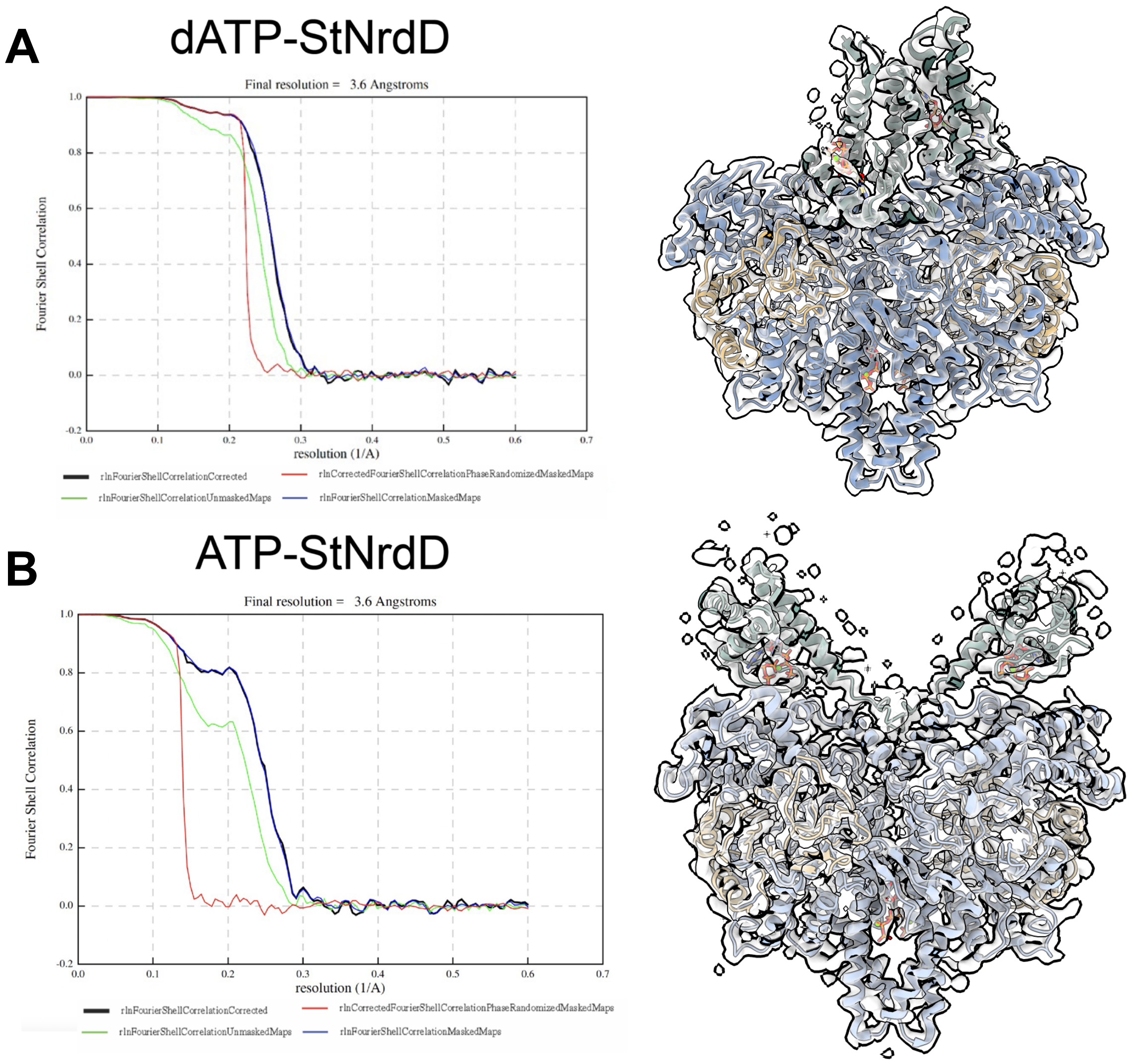

Fig. S7. FSC plots for maps of dATP- and ATP-StNrdD. A) FSC plots for masked (blue) and unmasked (green) map for dATP-StNrdD. The resolution is at 3.6 Å at a 0.143 cutoff. On the right, the dATP-bound StNrdD structure is displayed with the cryo-EM map, shown in transparent white volume (contoured at sdLevel 9, step size 1). B) FSC plots for masked (blue) and unmasked (green) map for ATP-StNrdD. The resolution is at 3.6 Å at a 0.143 cutoff. On the right, the ATP-bound StNrdD structure is displayed with the with cryo-EM map, shown in transparent white volume (contoured at sdLevel 9, step size 1).

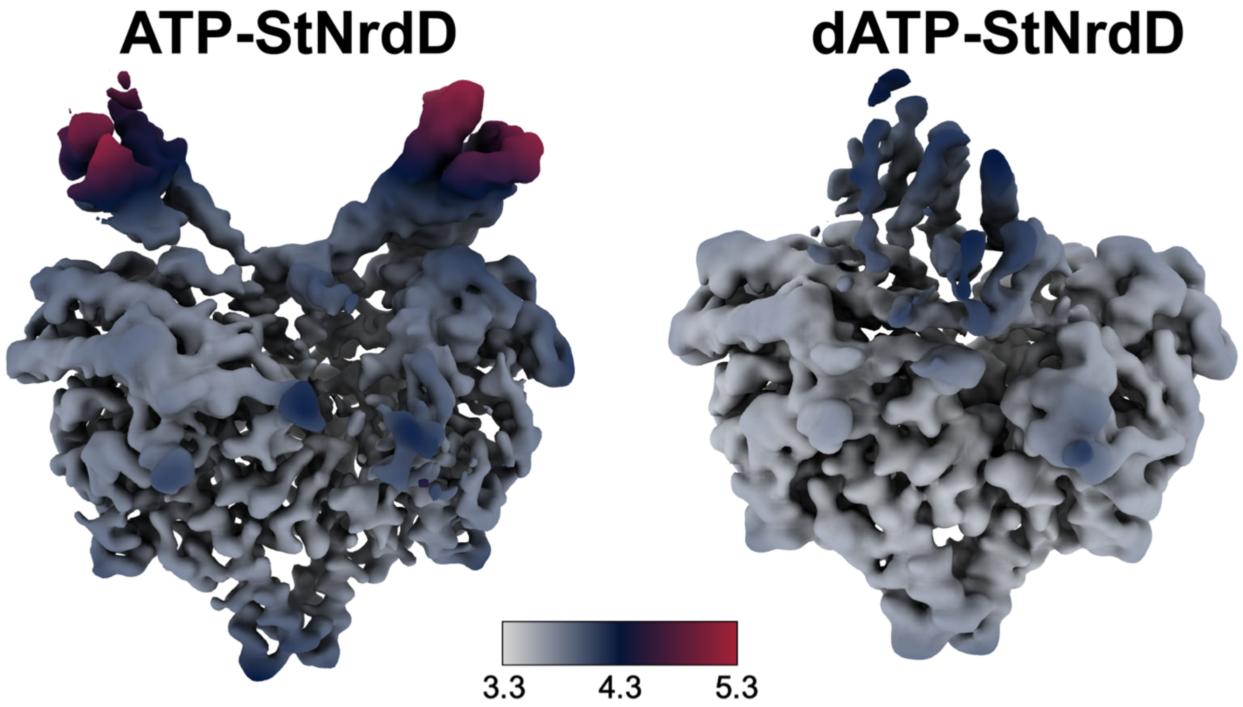

Fig. S8. Local resolution estimations for ATP-bound and dATP-bound StNrdD cryo-EM structures. The ATP-bound StNrdD structure was solved to 3.6-Å resolution, with local resolution ranging from ~5.2 Å in the cone domains to ~3.4 Å in the core. Resolution ranges are color coded from grey (3.5 Å) to red (5.2 Å), with the mid-range colored in navy (4.4 Å). The dATP-bound StNrdD structure was solved to 3.6-Å resolution, with local resolution ranging from ~4.4 Å in the cone domains to ~3.4 Å in the core. Resolution ranges are color coded from grey (3.4 Å) to red (4.4 Å), with the mid-range colored in navy (3.9 Å).

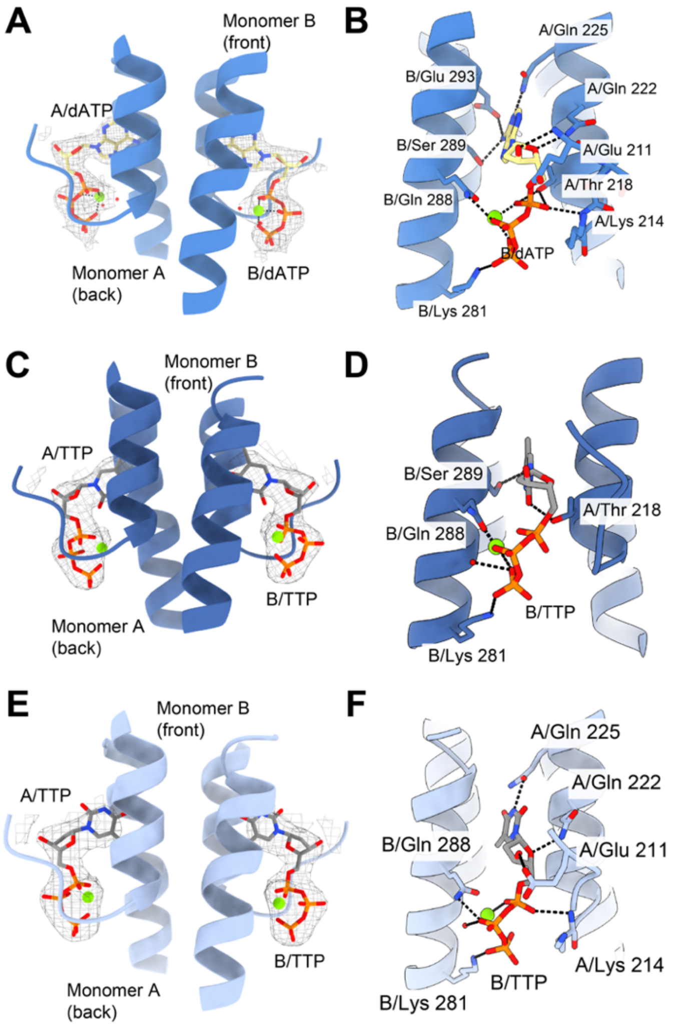

Fig. S9. **Omit map density and cryo-EM density for nucleotides bound in specificity sites in each StNrdD structure**. A) dATP-StNrdD crystal structure with $2F_o\text{-}F_c$ composite omit density (mesh contoured at 1 σ) for dATP bound in the specificity site. B) dATP-StNrdD crystal structure showing close interactions (4 Å or less, dashed black lines) made by dATP at the specificity site at the dimer interface. C) dATP-StNrdD cryo-EM structure with cryo-EM density (mesh contoured at sdLevel 9, step size 1) contoured around specificity effector TTP bound in the specificity site at the dimer interface. D) dATP-StNrdD cryo-EM structure showing close interactions (4 Å or less, dashed black lines) made by specificity effector TTP. E) ATP-StNrdD cryo-EM structure with cryo-EM density (mesh contoured at sdLevel 9, step size 1) around specificity effector TTP bound in the specificity site at the dimer interface. F) ATP-StNrdD cryo-EM structure showing close interactions (4 Å or less, dashed black lines) made by specificity effector TTP in the specificity site at dimer interface.

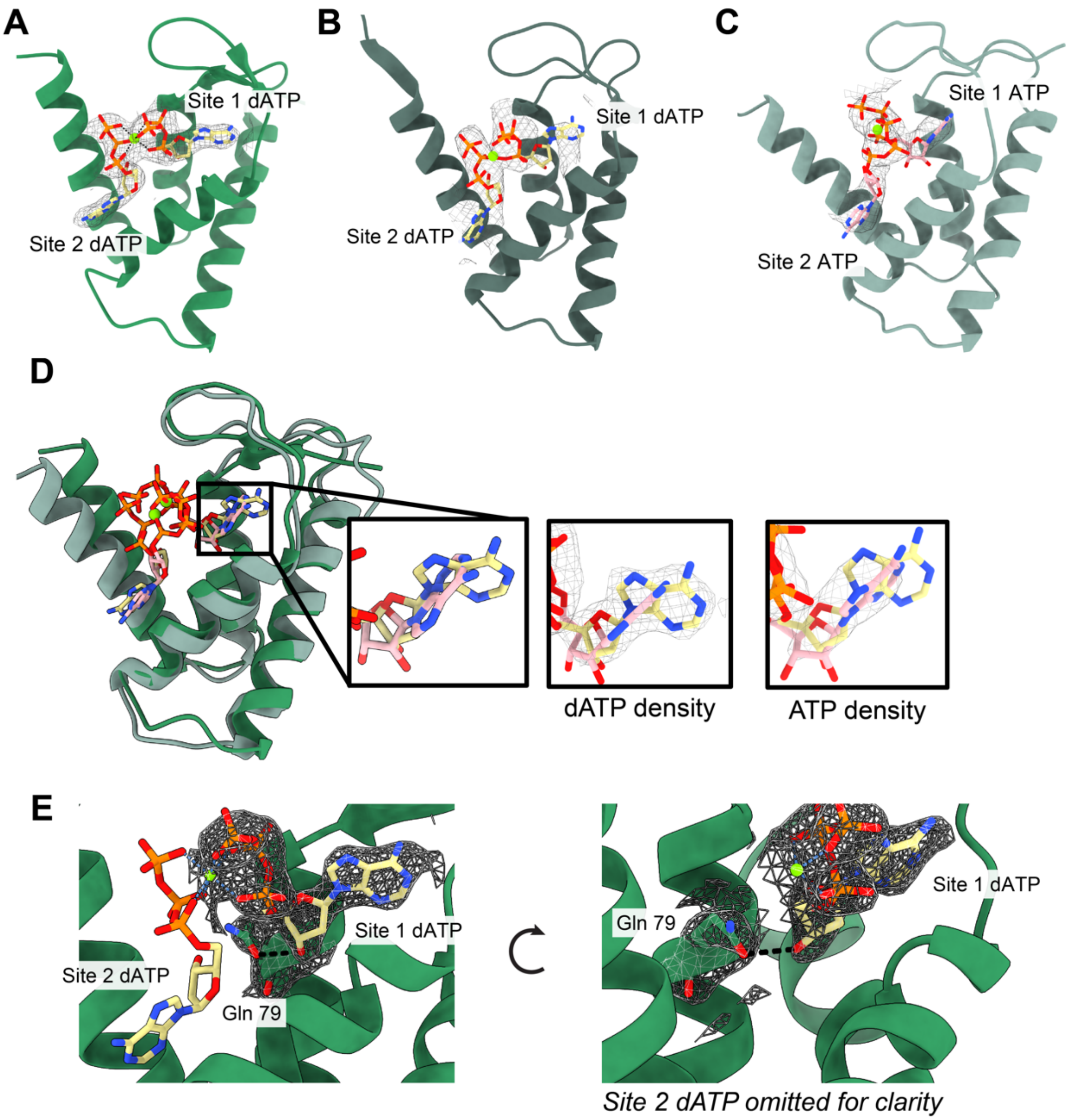

Fig. S10. Omit electron density and cryo-EM density for nucleotides bound in the cone domains of each StNrdD structure. A) dATP-bound StNrdD crystal structure showing $2F_o$-F_c composite omit density (mesh contoured at 1 σ) for activity effector dATP (yellow carbons) bound in the cone domain (green). B) dATP-StNrdD cryo-EM structure showing cryo-EM density (mesh contoured at sdLevel 9, step size 1) for activity effector dATP (yellow carbons) bound in the cone domain (dark green). C) ATP-StNrdD cryo-EM structure showing cryo-EM density (mesh contoured at sdLevel 9, step size 1) for activity effector ATP (pink carbons) bound in the cone domain (light green). D) Cone domain from the ATP-StNrdD cryo-EM structure (light green, ATP carbons in pink) aligned with cone domain from the dATP-StNrdD crystal structure (green, dATP carbons in yellow). Insets show close up views of the site 1 nucleotide ribose and base, protein is removed for clarity. For the dATP density inset, the $2F_o$-F_c composite omit density is contoured at 1 σ. For the ATP density, the cryo-EM density is contoured at sdLevel 9, step size 1. E) $2F_o$-F_c composite omit density showing interaction of Gln79 with Site 1 dATP from dATP-bound StNrdD crystal structure (mesh is contoured at 1 σ).

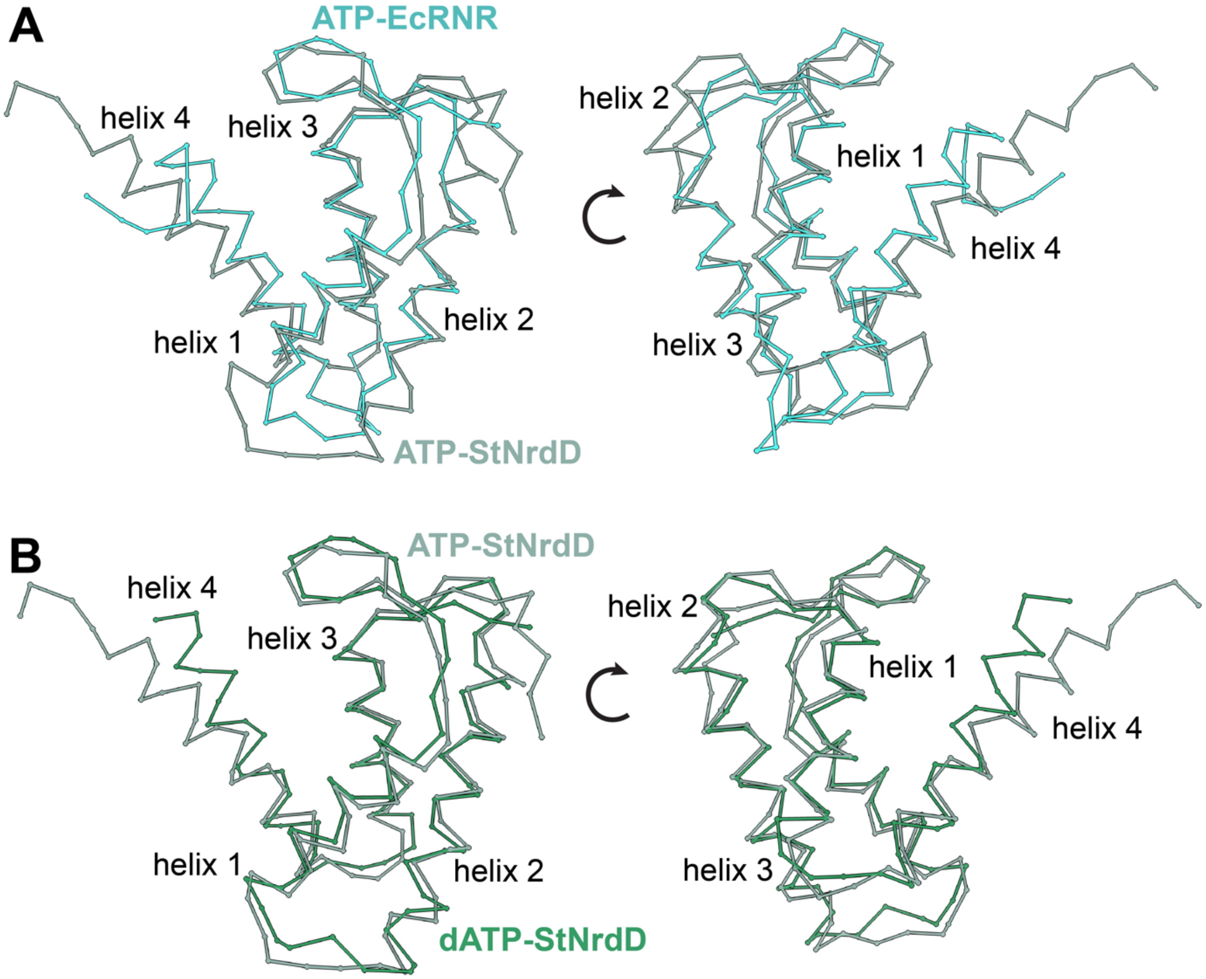

Fig. S11. **Class Ia and III cone domains have same architecture**. A) Alignment of the cone domains from ATP-bound StNrdD (light green) and ATP-bound EcRNR α_2-(ATP)$_2$ (turquoise). Cone domain helices are labeled. B) Alignment of the cone domains from the dATP-bound StNrdD crystallographic structure (green) and ATP-bound StNrdD (light green). Cone domain helices are labeled.

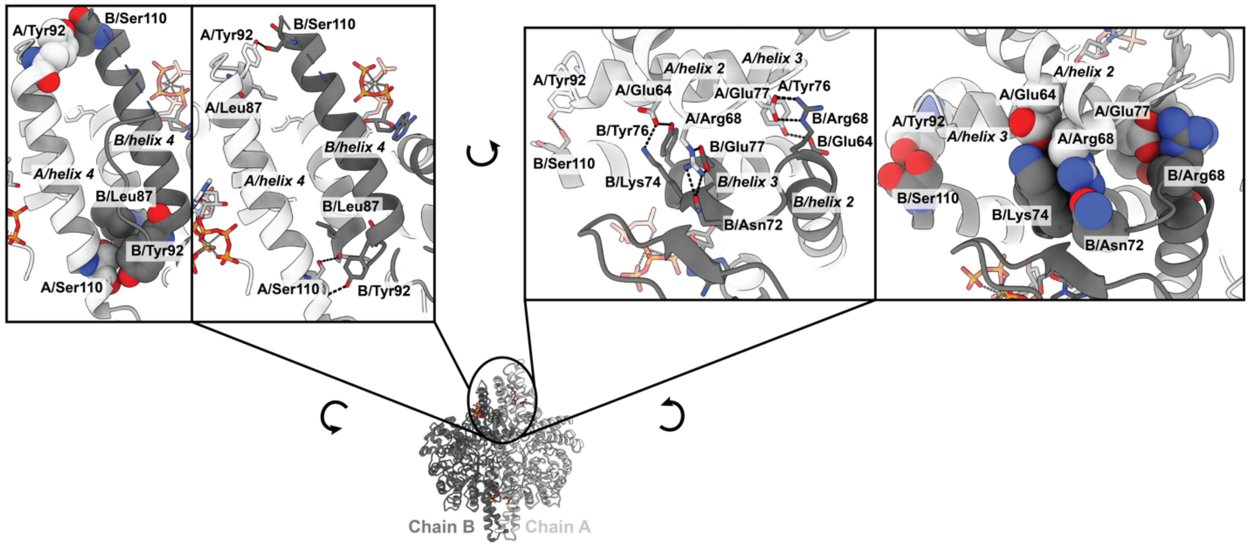

Fig. S12. dATP-bound StNrdD cone-cone interface. The dATP-bound StNrdD crystal structure has chain B colored in dark grey and chain A colored in light grey. Insets show close up views of the cone domain dimer interface, rotated from the full structure shown at the bottom. In each inset, the left image shows labeled interacting residues, with atoms in spheres, whereas the right image shows labeled interacting residues as sticks, with hydrogen bond interactions in black dotted lines. There are two major interacting sites along the cone-cone interface – the first along helix 4, the second along the points of helix 2 and 3.

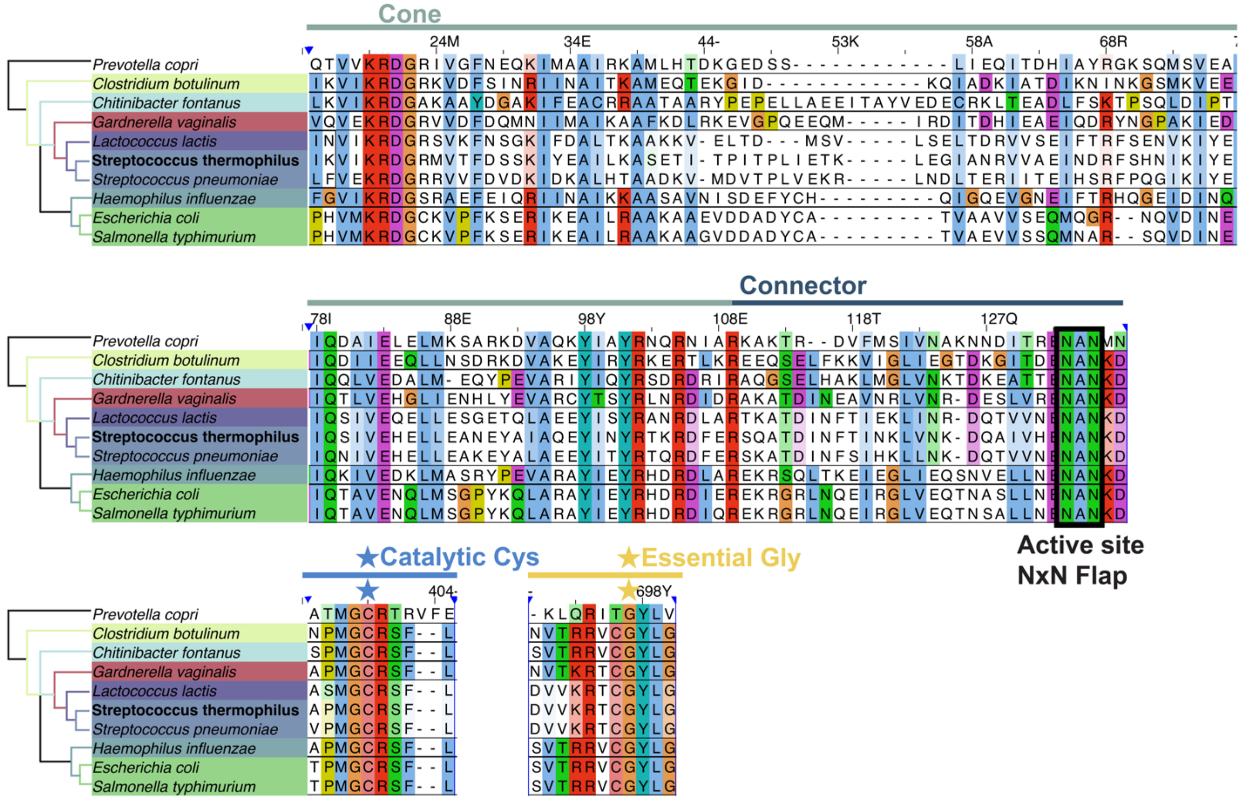

Fig. S13. Sequence alignment of class III RNR cone domains, connector regions, and active site residues. Ten class III RNR sequences are aligned using Clustal Omega and organized by phylogenetic order (neighbor joining tree, calculated with BLOSUM62, shown on the left, next to the organism name from which the class III RNR sequence was gathered). Residues are colored using the clustal format. The cone domain, connector (active site NxN flap indicated by black outline), region around the catalytic cysteine (indicated by blue star), and region around the essential glycine (indicated by yellow star) are shown. Numbering on top corresponds to the residue numbers from *S. thermophilus* NrdD (name highlighted in bold).

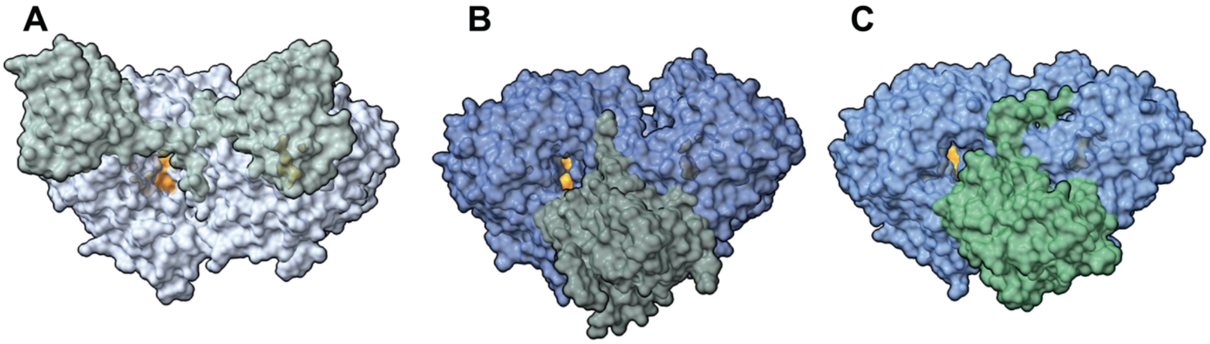

Fig. S14. The active sites are exposed in the StNrdD structures. A) Top-down view of ATP-bound StNrdD cryo-EM structure showing exposed active site residues (surface representation, core colored in light blue, cones in light green, active site residues in orange). B) Top-down view of dATP-bound StNrdD cryo-EM structure showing exposed active site residues (surface representation, core colored in dark blue, cones in dark green, active site residues in orange). C) Top-down view of dATP-bound StNrdD crystal structure showing exposed active site residues (surface representation, core colored in bright blue, cones in bright green, active site residues in orange).

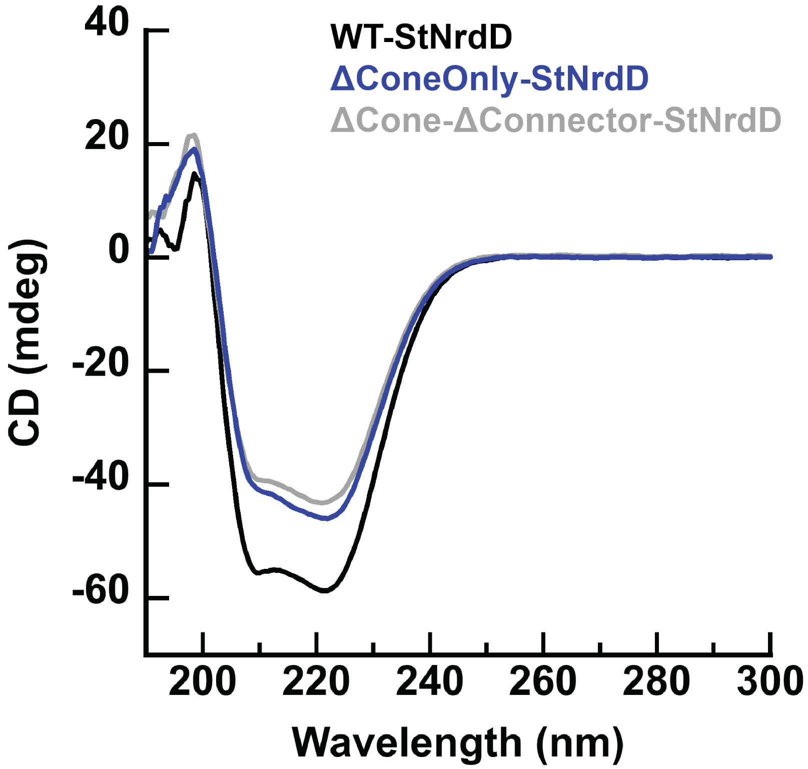

Fig. S15. Circular dichroism of StNrdD cone domains variants compared to wild type. Each sample consisted of 0.3 mg/mL protein in 50 mM sodium phosphate pH 7.6. Samples were measured using continuous scan mode from 190 nm to 300 nm at 22 °C, with a data pitch of 0.5 m, scan speed of 50 nm/min, CD scale of 200 mdeg/1.0 dOD, and with 3 accumulation replicates.

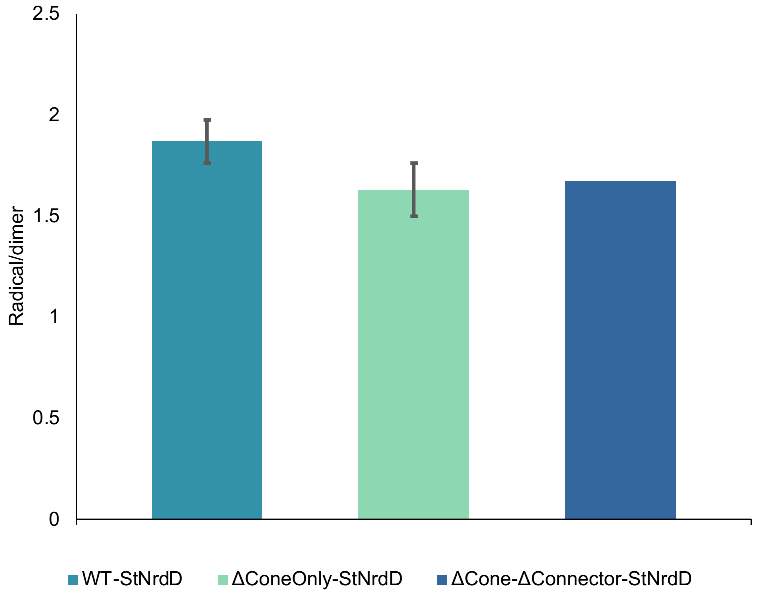

Fig. S16. Glycyl radical per dimer for StNrdD cone domain variants compared to wild type. Radical levels quantified by EPR using a Fremy's salt standard. Bars show the mean value over three replicates. Error bars show standard deviation. ΔCone-ΔConnector-StNrdD only had two replicates, so a standard deviation was unable to be calculated. 2 radicals per dimer is the maximum possible radical content.

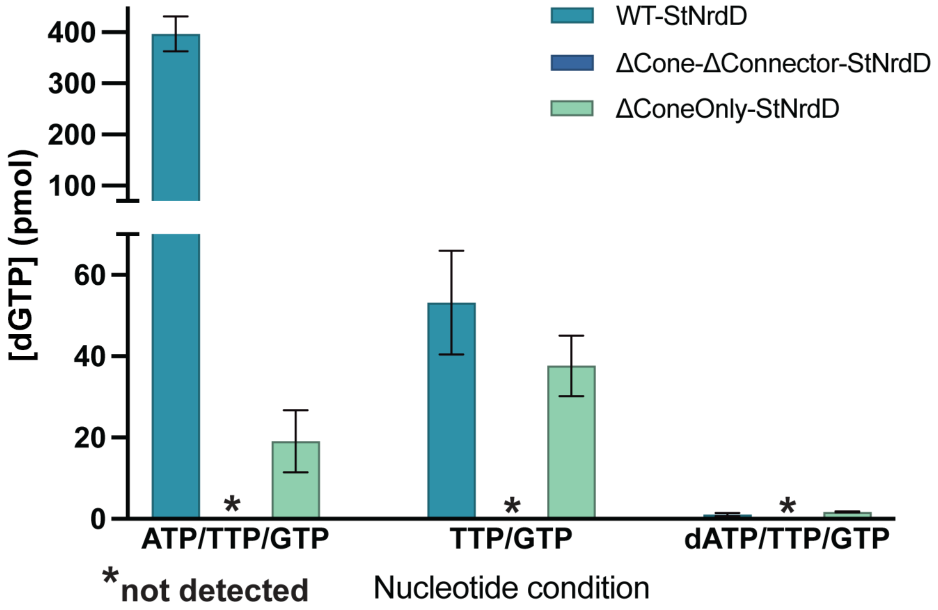

Fig. S17. Production of product (dGTP) by StNrdD wild type and variants in presence of activating (ATP) and inhibiting (dATP) nucleotide conditions. Activity assay data for wild type StNrdD, ΔCone-ΔConnector-StNrdD, and ΔConeOnly-StNrdD conducted in the presence of 1 mM TTP as the specificity effector, 1 mM GTP as the substrate, and 3 mM ATP or dATP as the activity effector, or no activity effector. Each bar represents average product concentration ± standard error of the mean for all replicates. The decrease in activity in presence of dATP/TTP/GTP and ATP/TTP/GTP over TTP/GTP alone is likely due to the competitive binding of ATP and dATP with GTP and TTP for the active site and specificity sites, respectively.

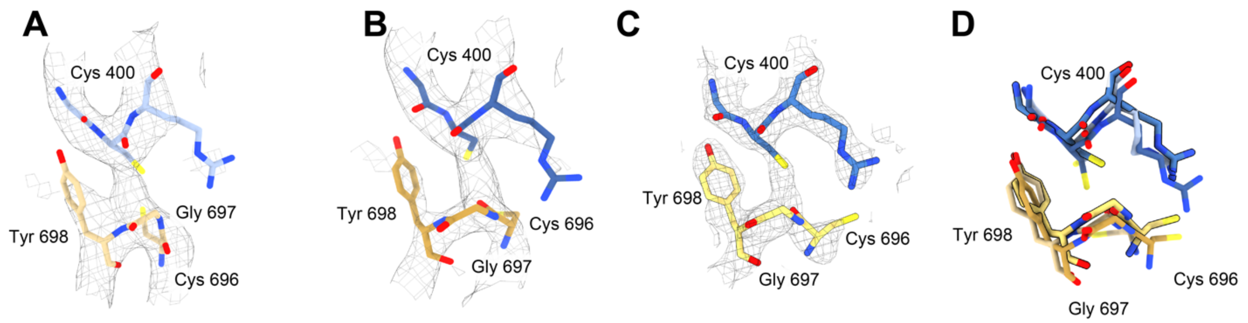

Fig. S18. Omit electron density and cryo-EM density for the active site residues in each StNrdD structure. A) Active site residues of the ATP-bound StNrdD cryo-EM structure. The Gly loop (colored in sand) and the Cys loop (colored in light blue). Cryo-EM density (contoured at sdLevel 9, step size 1) for residues is shown in mesh. B) Active site residues of the dATP-bound StNrdD cryo-EM structure. The Gly loop (colored in gold) and the Cys loop (colored in dark blue). Cryo-EM density (contoured at sdLevel 9, step size 1) for residues is shown in mesh. C) Active site residues of the dATP-bound StNrdD crystal structure. The Gly loop (colored in yellow) and the Cys loop (colored in blue). $2F_o$-F_c composite omit density contoured at 1 σ for the residues are shown in mesh. D) Overlay of active site residues of the ATP-bound (light blue and sand coloring), dATP-bound cryo-EM structure (dark blue and gold coloring), and dATP-bound StNrdD crystal structure (outlined in black with yellow and bright blue coloring).

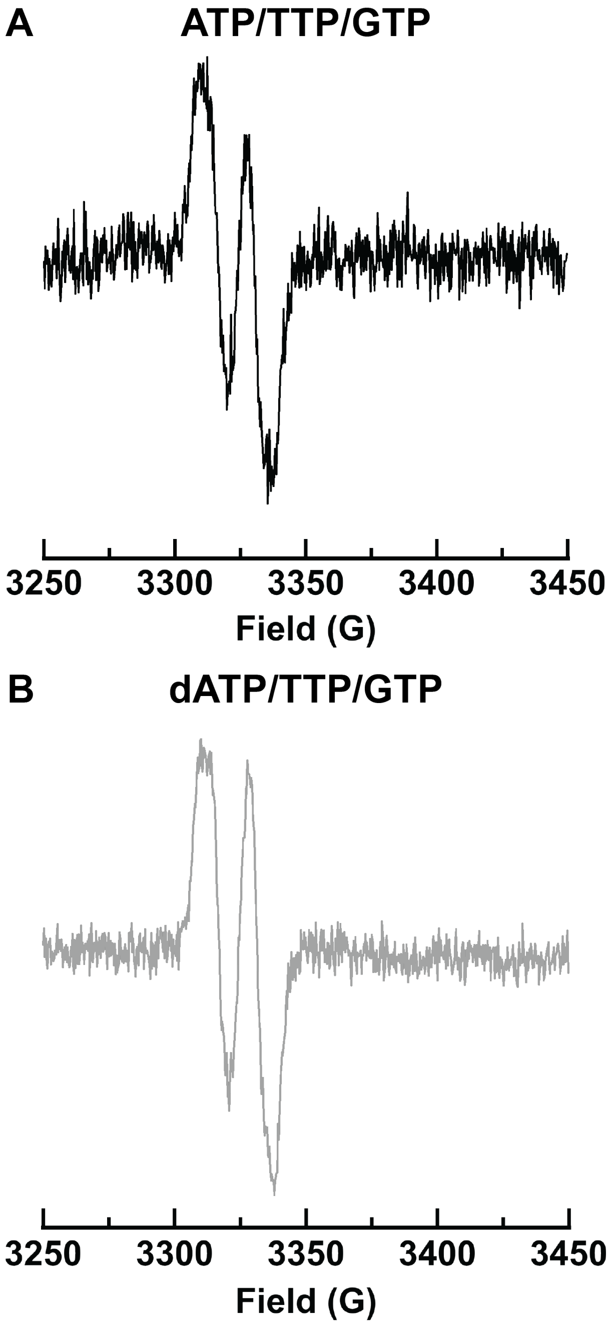

Fig. S19. Shape of the glycyl radical EPR spectrum in StNrdD is unaffected by substitution of allosteric activity effector ATP for dATP. 15mg/mL of StNrdD was pre-incubated for 2 min at 37 °C in 30 mM KCl, 30 mM Bicine, pH 7.6 supplemented with 10 mM $MgSO_4$ and either A) 3 mM ATP, 1 mM TTP, 1 mM GTP (ATP/TTP/GTP condition); or B) 3 mM dATP, 1 mM TTP, 1 mM GTP (dATP/TTP/GTP condition). EPR parameters were 80 K, 10 scans, modulation amplitude 3 G, center field 3350 G, sweep width 200 G, sweep time 21 s, microwave power 52 dB.

Table S1. StNrdD crystallography data collection and refinement statistics

	**dATP-StNrdD**
**Data collection**	
Space group	$P\ 2_12_12_1$
Cell dimensions	
a, *b*, *c* (Å)	90.580, 97.885, 189.202
α, β, γ (°)	90, 90, 90
Beamline	APS 24-ID-C
Wavelength (Å)	0.979
Resolution (Å)	48.94-2.60 (2.65-2.60)
Observed Reflections	532499 (87502)
Unique Reflections	52393 (8323)
R_{sym} or R_{merge}	0.133 (>0.801)
$\langle I/\sigma I \rangle$	15.98 (3.43)
$CC_{1/2}$ (%)	0.998 (0.925)
Completeness (%)	99.83 (98.90)
Redundancy	10.2 (10.5)
**Refinement**	
Resolution (Å)	2.60
No. reflections	52383
No. reflections R_{free}	2613
R_{work} / R_{free}	0.1729 / 0.2317
Model components	
Protein chains	2
Protein residues	1394 (of 1468)
Mg^{2+} ions	4
dATP molecules	6
SO_4 molecules	15
Zn ions	2
Water molecules	156
Average B factors (Å^2)	
Protein	47.15
Mg^{2+}	40.11
dATP	43.44
SO_4	69.72
Zn	91.40
Water	40.21
R.M.S deviations	
Bond length (Å)	0.002
Bond angle (°)	0.476
Ramachandran plot	
Favored (%)	96.68
Allowed (%)	3.25
Outliers (%)	0.07
Rotamer outliers (%)	0.91
Clashscore	4.16

Values for data in the highest resolution bin are shown in ()

Table S2. Cryo-EM data collection, refinement, and validation statistics

	ATP-bound StNrdD	dATP-bound StNrdD
**Data collection and processing**		
Microscope	Titan Krios G3i	Titan Krios G3i
Camera	Gatan K3	Gatan K3
Magnification	105,000X	105,000X
Voltage (kV)	300	300
Recording mode	counting	counting
Frames/Movies	30	30
Total Electron dose (e-/Å2)	51.35	51.43
Defocus range (μm)	-0.75 to -2.5	-0.75 to -2.5
Pixel size (Å)	0.8324	0.8324
Micrographs collected	10,794	21,675
Automation software	EPU	EPU
Total extracted particles	465,503	3,659,249
Refined particles	146,371	748,815
Symmetry imposed	none	none
Nominal Map Resolution (Å)	3.6	3.6
FSC threshold	0.143	0.143
masked/unmasked	3.6/3.6	3.6/3.6
Local resolution range (Å)	3.46-5.24	3.38-4.39
**Model composition**		
Non-hydrogen atoms	11376	11368
Residues		
Protein: Chain A	14-67, 70-119, and 138-732 (of 734)	17-118, and 136-732 (of 734)
Protein: Chain B	14-119, and 138-730 (of 734)	12-20, 25-118, 138-732 (of 734)
ATP	4	0
dATP	0	4
Zn^{2+}	1	2
Mg^{2+}	4	4
Water molecules	4	4
**Validation**		
MapCC (mask/box)	0.79/0.77	0.83/0.80
Mean CC for ligands	0.70	0.83
R.m.s deviations		
Bond lengths (Å)	0.013	0.003
Bond angles (°)	1.114	0.566
MolProbity score	2.90	2.41
Clashscore (all atom)	19.79	11.94
Rotamer outliers (%)	6.12	3.89

Ramachandran plot		
Favored (%)	91.43	94.66
Allowed (%)	8.57	5.34
Outliers (%)	0	0
CaBLAM outliers (%)	3.48	2.32

Table S3. Primers used for mutagenesis experiments.

**Primer**	**Sequence**
ΔCone-ΔConnector forward	GGCCTGGTGCCGCGCCATAGTGATTTAT
ΔCone-ΔConnector reverse complement	TTGTGTATTGTATAAATCACTATCGCGCG
ΔConeOnly forward	TCTCAGGCTACTGATATCAATTTCACCATTAACAAA CTTGTTAATAAAGATCAGGC
ΔConeOnly reverse complement	ATGGCTGCCGCGCGGCAC
